# Soil macrofauna communities in Brazilian land-use systems

**DOI:** 10.3897/BDJ.12.e115000

**Published:** 2024-01-15

**Authors:** George G. Brown, Wilian C Demetrio, Quentin Gabriac, Amarildo Pasini, Vanesca Korasaki, Lenita J. Oliveira, Julio C.F. dos Santos, Eleno Torres, Paulo R. Galerani, Dionisio L. P. Gazziero, Norton P. Benito, Daiane H. Nunes, Alessandra Santos, Talita Ferreira, Herlon S. Nadolny, Marie L. C. Bartz, Wagner Maschio, Rafaela T. Dudas, Mauricio R. G. Zagatto, Cintia C. Niva, Lina A. Clasen, Klaus D. Sautter, Luis C.M. Froufe, Carlos Eduardo S. Seoane, Aníbal de Moraes, Samuel James, Odair Alberton, Osvaldino Brandão Júnior, Odilon Saraiva, Antonio Garcia, Elma Oliveira, Raul M. César, Beatriz S. Corrêa-Ferreira, Lilianne S. M. Bruz, Elodie da Silva, Gilherme B. X. Cardoso, Patrick Lavelle, Elena Velásquez, Marcus Cremonesi, Lucília M. Parron, Amilton J. Baggio, Edinelson Neves, Mariangela Hungria, Thiago A. Campos, Vagner L. da Silva, Carlos B. Reissmann, Ana C. Conrado, Jean-Pierre D. Bouillet, José L. M. Gonçalves, Carolina B. Brandani, Ricardo A. G. Viani, Ranieri R. Paula, Jean-Paul Laclau, Clara P Peña-Venegas, Carlos Peres, Thibaud Decaëns, Benjamin Pey, Nico Eisenhauer, Miguel Cooper, Jérôme Mathieu

**Affiliations:** 1 Embrapa Florestas, Colombo, Brazil Embrapa Florestas Colombo Brazil; 2 UFPR, Curitiba, Brazil UFPR Curitiba Brazil; 3 ESALQ-USP, Piracicaba, Brazil ESALQ-USP Piracicaba Brazil; 4 Private, Saleilles, France Private Saleilles France; 5 Universidade Estadual de Londrina, Londrina, Brazil Universidade Estadual de Londrina Londrina Brazil; 6 Universidade do Estado de Minas Gerais, Frutal, Brazil Universidade do Estado de Minas Gerais Frutal Brazil; 7 Embrapa Soja, Londrina, Brazil Embrapa Soja Londrina Brazil; 8 Embrapa, Brasília, Brazil Embrapa Brasília Brazil; 9 Embrapa Recursos Genéticos, Brasília, Brazil Embrapa Recursos Genéticos Brasília Brazil; 10 Instituto Federal Catarinense, Camboriu, Brazil Instituto Federal Catarinense Camboriu Brazil; 11 Universidade Federal do Paraná, Curitiba, Brazil Universidade Federal do Paraná Curitiba Brazil; 12 CARE-Bio, Idanha-a-Nova, Portugal CARE-Bio Idanha-a-Nova Portugal; 13 DungTech Biofertilizantes Ltda, Piracicaba, Brazil DungTech Biofertilizantes Ltda Piracicaba Brazil; 14 Embrapa Cerrados, Planaltina, Brazil Embrapa Cerrados Planaltina Brazil; 15 University of Copenhagen, Copenhagen, Denmark University of Copenhagen Copenhagen Denmark; 16 Uniandrade, Curitiba, Brazil Uniandrade Curitiba Brazil; 17 Maharishi University, Fairfield, United States of America Maharishi University Fairfield United States of America; 18 Universidade Paranaense, Umuarama, Brazil Universidade Paranaense Umuarama Brazil; 19 Fatec, Araçatuba, Brazil Fatec Araçatuba Brazil; 20 Université Pierre et Marie Curie (Paris 6), Paris, France Université Pierre et Marie Curie (Paris 6) Paris France; 21 Universidad Nacional de Colombia, Palmira, Colombia Universidad Nacional de Colombia Palmira Colombia; 22 Federal University of Paraná (UFPR), Curitiba, Brazil Federal University of Paraná (UFPR) Curitiba Brazil; 23 Universidad de la República, Montevidéo, Uruguay Universidad de la República Montevidéo Uruguay; 24 CIRAD, Montpellier, France CIRAD Montpellier France; 25 Texas A&M AgriLife, Amarillo, United States of America Texas A&M AgriLife Amarillo United States of America; 26 Universidade Federal de São Carlos, Araras, Brazil Universidade Federal de São Carlos Araras Brazil; 27 Université du Québec, Chicoutimi, Canada Université du Québec Chicoutimi Canada; 28 SINCHI, Letícia, Colombia SINCHI Letícia Colombia; 29 University of East Anglia, Norwich, United Kingdom University of East Anglia Norwich United Kingdom; 30 CEFE, Univ Montpellier, CNRS, EPHE, IRD, Univ Paul Valéry Montpellier 3, Montpellier, France CEFE, Univ Montpellier, CNRS, EPHE, IRD, Univ Paul Valéry Montpellier 3 Montpellier France; 31 Université de Toulouse, Toulouse, France Université de Toulouse Toulouse France; 32 German Centre for Integrative Biodiversity Research (iDiv) Halle-Jena-Leipzig, Leipzig, Germany German Centre for Integrative Biodiversity Research (iDiv) Halle-Jena-Leipzig Leipzig Germany; 33 Leipzig University, Leipzig, Germany Leipzig University Leipzig Germany; 34 Sorbonne Université, Paris, France Sorbonne Université Paris France

**Keywords:** soil macroinvertebrates, biodiversity, Atlantic forest, agriculture, land-use impacts, bioindicators

## Abstract

**Background:**

Soil animal communities include more than 40 higher-order taxa, representing over 23% of all described species. These animals have a wide range of feeding sources and contribute to several important soil functions and ecosystem services. Although many studies have assessed macroinvertebrate communities in Brazil, few of them have been published in journals and even fewer have made the data openly available for consultation and further use. As part of ongoing efforts to synthesise the global soil macrofauna communities and to increase the amount of openly-accessible data in GBIF and other repositories related to soil biodiversity, the present paper provides links to 29 soil macroinvertebrate datasets covering 42 soil fauna taxa, collected in various land-use systems in Brazil. A total of 83,085 georeferenced occurrences of these taxa are presented, based on quantitative estimates performed using a standardised sampling method commonly adopted worldwide to collect soil macrofauna populations, i.e. the TSBF (Tropical Soil Biology and Fertility Programme) protocol. This consists of digging soil monoliths of 25 x 25 cm area, with handsorting of the macroinvertebrates visible to the naked eye from the surface litter and from within the soil, typically in the upper 0-20 cm layer (but sometimes shallower, i.e. top 0-10 cm or deeper to 0-40 cm, depending on the site). The land-use systems included anthropogenic sites managed with agricultural systems (e.g. pastures, annual and perennial crops, agroforestry), as well as planted forests and native vegetation located mostly in the southern Brazilian State of Paraná (96 sites), with a few additional sites in the neighbouring states of São Paulo (21 sites) and Santa Catarina (five sites). Important metadata on soil properties, particularly soil chemical parameters (mainly pH, C, P, Ca, K, Mg, Al contents, exchangeable acidity, Cation Exchange Capacity, Base Saturation and, infrequently, total N), particle size distribution (mainly % sand, silt and clay) and, infrequently, soil moisture and bulk density, as well as on human management practices (land use and vegetation cover) are provided. These data will be particularly useful for those interested in estimating land-use change impacts on soil biodiversity and its implications for below-ground foodwebs, ecosystem functioning and ecosystem service delivery.

**New information:**

Quantitative estimates are provided for 42 soil animal taxa, for two biodiversity hotspots: the Brazilian Atlantic Forest and Cerrado biomes. Data are provided at the individual monolith level, representing sampling events ranging from February 2001 up to September 2016 in 122 sampling sites and over 1800 samples, for a total of 83,085 ocurrences.

## Introduction

Worldwide, soils may host from 40 to 60% of the world’s species (FAO 2020, Anthony et al. 2023) and are still considered one of the main biotic frontiers (Geisen et al. 2019). Neotropical regions are particularly biodiverse and host numerous endangered hotspots (Myers et al. 2000). Furthermore, neotropical soils can support a megadiverse biota that drive ecosystem functioning affecting the delivery of essential ecosystem services, including crop production, biological pest control, nutrient cycling, seed dispersal, pollination, water storage and availability, pedogenesis, soil erosion control and carbon sequestration (Brown et al. 2015). Amongst these are the incredibly diverse soil microorganisms (Torsvik and Øvreås 2002, Tedersoo et al. 2014), as well as the soil fauna, which are usually classified according to their size into microfauna (microscopic animals < 0.1 mm in diameter, mainly nematodes, tardigrades and rotifers), mesofauna (small animals from 0.1 to 2 mm in diameter, mainly mites and springtails), macrofauna (larger invertebrates visible to the naked eye; Ruiz et al. (2008)) and the megafauna, i.e. vertebrates like moles, small rodents and some snakes, for instance (Swift et al. 1979).

The soil and surface-litter dwelling macrofauna include the larger easily-visible invertebrate taxa (Ruiz et al. 2008) and represent from 23-27% of all described species (Decaëns et al. 2006, Anthony et al. 2023). Amongst these are more than 40 major taxonomic groups, such as the well-known ants (Formicidae), termites (Isoptera), earthworms (Crassiclitellata), beetles and their larvae (Coleoptera), millipedes (Diplopoda), centipedes (Chilopoda), fly larvae (Diptera), spiders (Araneae), scorpions (Scorpiones), cicadas (Hemiptera, Auchenorrhyncha), crickets (Orthoptera), woodlice (Isopoda) and cockroaches (Blattaria) (Brown et al. 2018, Lavelle et al. 2022b). However, a large number of less well-known taxa are also part of the soil macrofauna (see Table 1), though they don’t tend to be so commonly collected with the more widely used sampling methods (Gongalsky 2021).

Considering this wide range of taxa and the taxonomic impediment afflicting many soil-dwelling animals (Decaëns et al. 2006), it not surprising that the overall richness of soil macroinvertebrates remains poorly known, particularly in the tropics (Cameron et al. 2019, Guerra et al. 2020). This taxonomic richness also implies a variety of morphological and functional adaptations developed to live in the soil, so that the soil macrofauna have evolved into a wide range of “functional groups”, typically characterised by their food sources (Lavelle 1996). These include the detritivores, coprophages, xylophages, predators, parasites, phytophages, fungus-growers and fungivores, geophages, omnivores and the ecosystem engineers (Lavelle et al. 1997, Potapov et al. 2022b; see Table 1). Hence, their activities contribute to several important ecosystem services to human beings (Lavelle et al. 2006, Brown et al. 2018). Furthermore, because of their high sensitivity to land-use management and changes in vegetation cover and soil properties, they are frequently used as bioindicators of disturbance (e.g. Paoletti et al. (1996), Paoletti et al. (2007), Lavelle et al. (2021)) and of soil quality and ecosystem service delivery (e.g. Rousseau et al. (2013), Franco et al. (2017), Bünemann et al. (2018), Velasquez and Lavelle (2019), Sofo et al. (2020)).

The Atlantic Forest and the Cerrado (Brazilian Savannah) biomes are two global biodiversity hotspots in Brazil (Myers et al. 2000) highly threatened by urbanisation and agricultural expansion (Strassburg et al. 2017, SOSMA/INPE 2021). These biomes (particularly the Cerrado) were also proposed by Guerra et al. (2022) as priority areas for soil biodiversity conservation. Combined, these regions are home to more than 70% of Brazil’s population (IBGE 2023) and provide essential ecosystem services, particularly related to water availability and storage. Many of Brazil’s main rivers are born in these biomes and the Guarani Aquifer, one of the largest in the world lies underneath them (Sindico et al. 2018). However, the richness of the soil fauna living in these biomes remains vastly unknown (Lewinsohn et al. 2005, Brown et al. 2015), despite the large number of active taxonomists in the region (Lewinsohn 2005) and the relatively large sampling effort (Araujo et al. 2020, Mathieu et al. 2022). This extends to the speciose soil macroinvertebrates, in which a large number of new taxa have been described over the last 10 years (CTFB 2023), with an additional large number of new species still to be found and described (Brown et al. 2015).

Brazil is the country with the highest number of sampling sites regarding soil macrofauna populations (Mathieu et al. 2023b), but many of the studies have not been published in peer-reviewed journals (Araujo et al. 2020) and almost none of them has provided open-access to the primary data collected on the soil fauna and environmental/soil attributes (see notable exceptions in Demetrio et al. (2020), Demetrio et al. (2021), Lavelle et al. (2022a). Furthermore, little quantitative data are available in GBIF in terms of sampling events involving soil macroinvertebrate communities in the tropics, so a special call of SoilBON and the Colorado State University with support from GBIF (Guerra and Wall 2023) represented a unique opportunity to provide additional macrofauna data to GBIF. Increasing access to primary data allows for better comparability between studies and for an improved understanding of soil macroinvertebrate communities in Neotropical land-use systems and vegetation covers. By quantifying the populations of various invertebrate taxa in different land uses, inferences can be made regarding the sustainability of land management practices, as well as of their potential contribution to ecosystem service provisioning (Velasquez and Lavelle 2019, Lavelle et al. 2021), such as through the use of various foodweb models (Potapov 2022).

## General description

### Purpose

In the present paper, we provide a suite of quantitative datasets on soil macrofauna communities collected using standard methods, in various Brazilian natural and anthropogenic ecosystems. The work was developed as part of the goals of two internationally-funded projects, aiming to synthesise the results available on soil macroinvertebrate communities in Neotropical biomes and another at the global level (Mathieu et al. 2022).

### Additional information

Our efforts focused on collating soil macrofauna and soil analysis metadata, with a few sites in the Cerrado, but with most of them in the Atlantic Forest biome, where most of the studies on soil macrofauna have been made using standard sampling methods (Mathieu et al. 2023b). The datasets cover 122 sites in 23 Brazilian counties, most of them (96) in the southern State of Paraná, with five sites in Santa Catarina and 21 in the south-eastern State of São Paulo (Table 2). The combined 29 datasets include a total of 1,855 individual samples (monoliths = sampling events), with 83,085 occurrences from a wide range of land uses, for example, native vegetation (grassland, forests), forestry plantations with various native (*Araucaria*, *Inga*, *Myrsine*) and exotic (*Acacia*, *Eucalyptus*, *Pinus*) tree species, all types of integrated management systems (agrosilvopastoral, silvopastoral, agropastoral and agroforestry), orchards, pastoral systems, urban areas and a variety of agricultural crops (mainly annual grain crops like soybean, maize and wheat).

The information in the datasets included data on macrofauna abundance (number of individuals in all datasets and biomass in the majority of the datasets) of 42 soil macrofauna taxa (Table 1). When the taxa were not encountered, 0 was used as the value for the monolith data. This does not mean that the animal was necessarily absent from the site, but rather that it was not found with the sampling effort performed (determined by the number of monoliths and the number of times the site was sampled), using the standard sampling methodology for soil macrofauna communities (TSBF), as specified in the Methods section. Most of the datasets also include a number of soil attributes, derived from chemical (pH, exchangeable acidity, Carbon, Phosphorus, Calcium, Magnesium, Potassium, Aluminium, Cation Exchange Capacity, Base Saturation in most cases and, rarely, total Nitrogen) and physical analysis (particle size distribution; i.e. sand, silt, clay percentages and, infrequently, soil moisture and bulk density) of soil samples taken at the same sites, generally from the same monoliths.

## Project description

### Title

The relationship between soil macrofauna biodiversity and ecosystem services delivery across land-use systems in Neotropical rainforest biomes (FaunaServices)

### Personnel

The FaunaServicesproject PIs are Jerome Mathieu (Sorbonne Université, France) and Miguel Cooper (University of São Paulo, ESALQ campus, Brazil), assisted mainly by George G. Brown (Embrapa Forestry, Brazil), Wilian C. Demetrio (ESALQ-USP, Brazil) and Quentin Gabriac (France) for the gathering, processing and inclusion/submission of soil macrofauna data. Additional official project participants include Marie L.C. Bartz (CARE-BIO and University of Coimbra, Portugal), Lucília P. Vargas (Embrapa Forestry, Brazil), Carlos Peres (University of East Anglia, England), Thibaud Decaëns (CEFE-Université Montpellier, France), Benjamin Pey (Université de Toulouse, France) and Clara Peña Venegas (Sinchi, Colombia), though several other researchers from Brazil and abroad have been invited to contribute to the project goals.

### Study area description

The area of focus of the project is mainly the Atlantic Forest and Amazonia although a few datasets from other biomes of Brazil (e.g. Cerrado) have been included as well. In the current paper, we focus only on datasets from Brazil, but data are also available from other Amazonian countries (mainly Colombia, Peru and French Guyana).

### Design description

The taxonomic focus of the project is on the soil macroinvertebrates and their populations (abundance and biomass, when available), obtained using standardised quantitative measurements. Furthermore, additional soil physical and chemical data have been gathered in order to provide a more robust assessment of potential contributions of both the land-use systems and the soil fauna towards the delivery of various ecosystem services, focusing primarily on:

1. water percolation and retention in soils, calculated from soil properties with equations from soil science, in particular pedotransfer functions (Medeiros et al. 2014);
2. plant productivity, derived from satellite data;
3. nutrient cycling, including mainly phosphorus and nitrogen and cation mineralisation and availability;
4. organic matter stocks in the topsoil, calculated from soil C contents and bulk density;
5. biological control capacity, assessed using analysis of the foodweb structure of the macrofauna communities.

The project aims to provide key databases on lesser-known, under-represented soil taxa, that can be used for a variety of other studies in the future, relating soil biodiversity with ecosystem functioning and with various drivers (both natural and anthropogenic) of biodiversity at different geographical/temporal scales. Data on the abundance and biomass (when present) of 42 soil macrofauna taxa were prepared in standard excel datasheets using the standard template of Mathieu et al. (2023a). These were then modified in order to follow Darwin Core standard variables, with the assistance of the Brazilian node of GBIF (SiBBr). Soil analysis metadata and information on the land-use systems were also provided (see Table 3), although the template of Mathieu et al. (2023a) is much more extensive. The current datasets represent only around 25% of the available data on soil macrofauna communities from Brazil and a large number of additional datasets (see Suppl. material 1) have been prepared, but these have not yet been adapted to the Darwin Core.

### Funding

The project is funded by a joint synthesis call between France and Brazil entitled "Biodiversity in the Neotropical Realm", by CESAB/CEBA-FAPESP/CNPq, facilitated through the French FRB (Foundation for Research on Biodiversity) and the SinBiose programme of CNPq (Conselho Nacional de Desenvolvimento Científico e Tecnológico). On the French side, the call is funded by CESAB and CEBA (Center for the Study of Biodiversity in Amazonia) and on the Brazilian side by the CNPq (post-doctoral grant to WCD) and FAPESP (Fundação de Amparo à Pesquisa do Estado de São Paulo).

## Sampling methods

### Study extent

All sites (Table 2) were sampled using the standard method developed by the Tropical Soil Biology and Fertility Programme, published in Anderson and Ingram (1993) and also further proposed for soil macrofauna sampling by ISO (2011). This method was devised by Lavelle (1988) as a means of quantitatively estimating soil macroinvertebrate taxa at a particular site and moment in time (sampling event) and represents the most widely used method worldwide to quantitatively assess soil macrofauna communities with a single sweep (Mathieu et al. 2022). Clearly, more efficient methods exist to quantify populations of some of the soil and litter-dwelling taxa individually (Gongalsky 2021) and, although it is not the best or ideal method to evaluate the populations of social insects like the soil dwelling and/or nest-building Hymenopterans (Formicidae, Vespidae, Apiidae) or termites (Bignell 2009), it still represents the most efficient, cheap (cost-effective) and simple-to-apply method to obtain quantitative estimates of the soil macrofauna community as a whole.

### Sampling description

**Figure 1.**
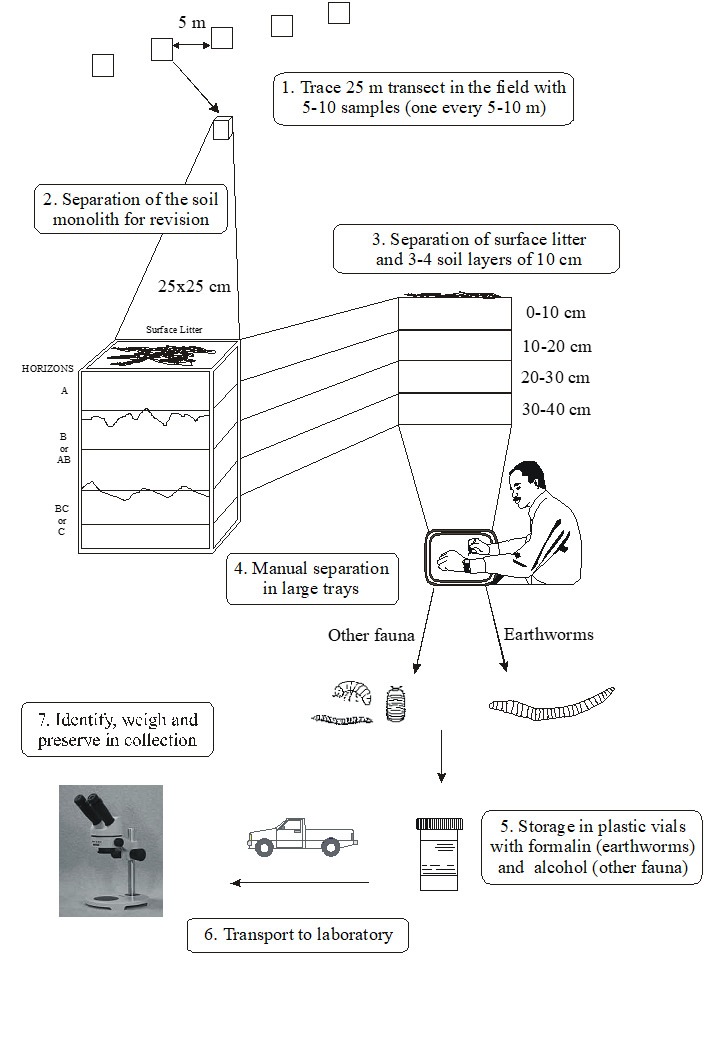
Step-by-step diagram illustrating the handsorting method of the TSBF (Tropical Soil Biology and Fertility) Programme, standardised by ISO (2011) and used to quantitatively sample soil macroinvertebrate communities. Samples are located on a transect or grid, at distances of at least 10 m and preferably 20+ m from each other and a soil monolith of variable depth (but usually up to 30 cm) is removed in depth increments (usually 10 cm thick) and placed into a plastic bag or bucket and subsequently sorted by hand, to remove all the soil macrofauna visible to the naked eye. The animals are fixed and stored in ethanol at approx. 80% and taken to be identified in the laboratory. All of the main taxa (Table 1) are quantified and their fresh biomass is estimated using a 0.0001 g balance. Figure from Brown et al. (2001).

**Figure 2.**
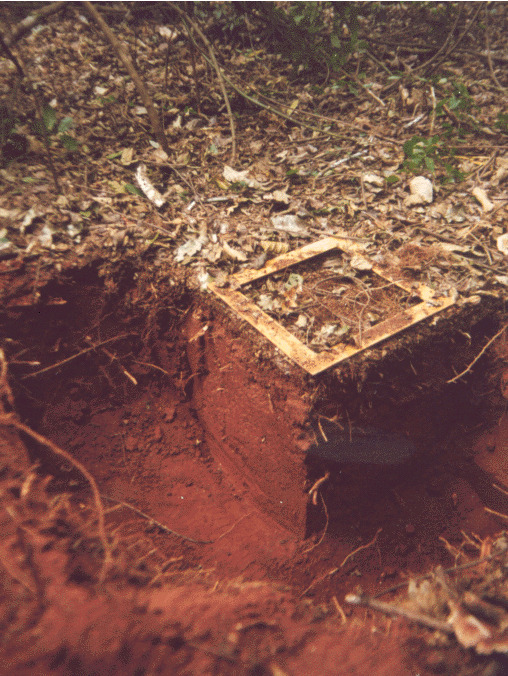
An example of a modification of the TSBF soil monolith sample from a semi-deciduous tropical forest, where an "L"-shaped hole is dug in front of the monolith to facilitate the removal of the soil layers of different depth increments. The quadrat on top has an internal area of 25 x 25 cm, which is used to mark the area from which the surface litter (when present) is removed for handsorting of the surface-dwelling macroinvertebrates. Photograph by George Brown.

**Figure 3.**
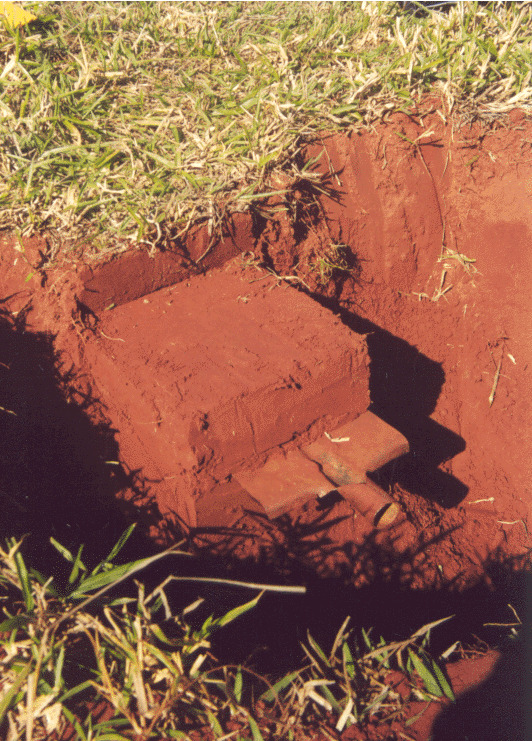
Image of a TSBF monolith sample from a pasture, where only one side of the monolith remains connected and where a flat straight spade is used to cut 10-cm layers of soil for handsorting. Photograph by George Brown.

**Figure 4.**
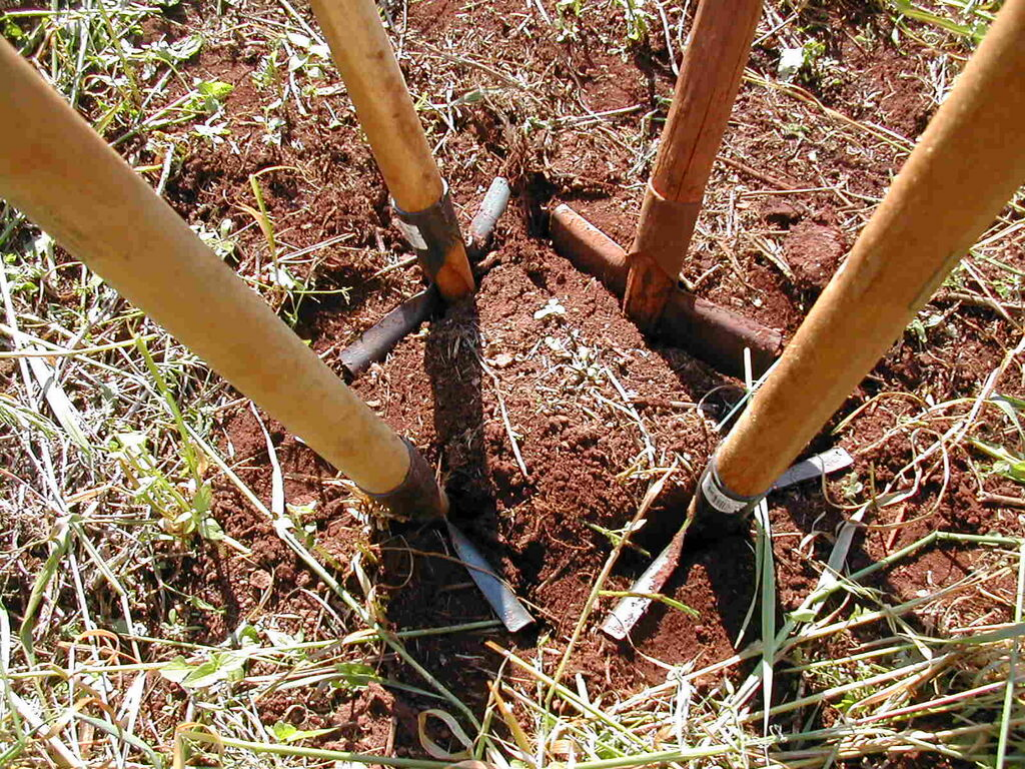
Isolation of a TSBF monolith, in which the sample is removed without digging around the hole. This is more effective when only the top 0-10 cm or, at most, the 0-20 cm layer is going to be sampled and handsorted. Photograph by George Brown.

**Figure 5.**
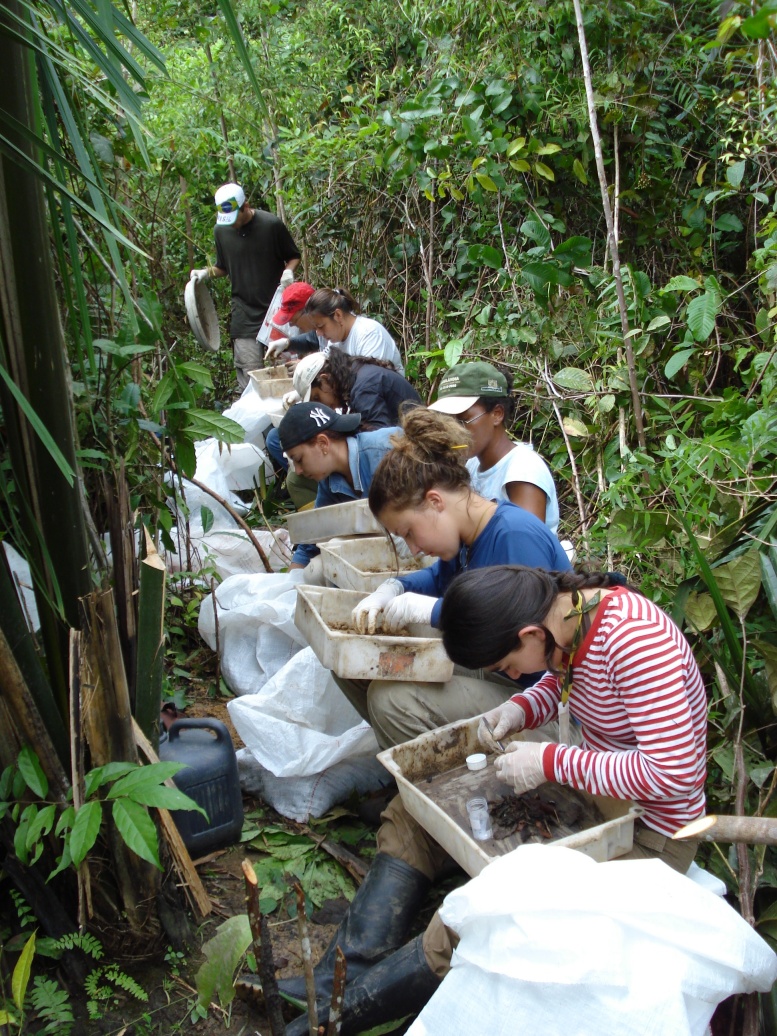
Handsorting of soil macrofauna samples taken in a regenerating forest near Pacajá, Pará, Brazil (May 2008). A small amount (at most two handfuls) of soil is placed into the large white plastic trays and is systematically manually sorted by removing the soil macroinvertebrates by hand or with the aid of small tweezers or even paint-brushes. The fauna are placed into small plastic vials containing ethanol at around 80%. Photograph by George Brown.

The TSBF method consists in handsorting individual monoliths of soil 25 x 25 cm in area, by first collecting the surface litter (when present) and then manually removing all invertebrates visible to the naked eye (Ruiz et al. 2008) present in the litter and in soil layers typically down to 30 cm (Figs 1, 2). Various modifications of the method have been used, with focus on more surface-dwelling fauna (restricting sampling to the 0-10 cm depth) and/or by removing the soil layers without physically isolating the monolith from the surrounding soil (Aquino 2001, Brown et al. 2001) (Figs 3, 4). The invertebrates removed by hand (or with the help of tweezers and sometimes paint-brushes) are placed into individually-labelled plastic vials (Fig. 5) with 70-99% ethanol. Higher concentrations are useful for posterior DNA extraction if molecular analyses are planned. Originally, earthworms were placed in dilute formalin solution (5-10%), but, due to its carcinogenic properties (and difficulty in posterior DNA extraction), they are usually now killed and fixed in 80% ethanol. The fauna are identified in the laboratory with the aid of a stereomicroscope and grouped into the major taxa (Table 1) and, subsequently, quantified and weighed (fresh biomass of each major taxa altogether, after leaving to dry in paper towels for at most a couple of minutes). To facilitate comparisons between different studies, results are usually presented as number of individuals m^-2^, although, in the present datasets, they are all given as number of individuals monolith^-1^ (sample^-1^). Two useful videos visually demonstrating the method step-by-step are available on YouTube in English (https://youtu.be/PkZuW0rJtZI) and Spanish (https://www.youtube.com/watch?v=BZHbNLMpLRs).

Additional samples are usually taken from the same monoliths or from their surroundings (e.g. individual or bulk samples) for soil chemical and physical analyses. Individual soil samples are preferred as they can then be used to interpret local variability in abundance values of the various taxa. Samples are frequently taken at different depth increments (usually of 10 cm thickness) as for the soil fauna, but are usually reported as a mean of all depth layers analysed. The soil variables (Table 3) include the standard list of attributes analysed in Brazilian soil analysis laboratories, for example, pH, organic C (Walkley-Black), available P, Ca, K, Mg and Al contents, Exchangeable acidity, Cation Exchange Capacity and Base Saturation, as well as particle size distribution (% sand, silt and clay). In some sites, total N (Kjehdhal), soil moisture and bulk density were also measured. All measurements followed standard methods described in Marques and Motta (2003) and/or Teixeira et al. (2017).

### Quality control

All datasets were prepared as Excel spreadsheets using a standard template (Mathieu et al. 2023a), which has the taxa in columns and the sites in rows and includes metadata on a large number of environmental and human management-related variables. Since the GBIF Darwin Core focuses mostly on the taxonomic backbone and data related to abundance of the taxa encountered (sampling events), a macro was prepared in Excel to facilitate the relocation of data on taxa from columns to rows. A similar process was done for the soil chemistry and physics data, which was included in a separate file (measurements table). Unfortunately, much of the environmental and management metadata was not transferable to the standard GBIF templates due to the lack of descriptor variables for them. Nevertheless, some of the more important ones such as vegetation cover and land use were included.

## Geographic coverage

### Description

The Atlantic Forest biomes spreads from north-eastern Brazil down to the southernmost State of Brazil (Rio Grande do Sul), although the data in the present dataset are mainly from the southern State of Paraná (n = 24 datasets), with only five having data from the neighbouring States of São Paulo (n = 4 datasets) and Santa Catarina (n = 1 dataset). The Atlantic Forest includes several vegetation types (Oliveira‐Filho and Fontes 2006), with the main representatives being:

1. Lowland and Montane rainforests (generally broadleaf and evergreen), particularly prevalent along the coastal plain, the piedmont and the Atlantic Coastal Mountain Range (“Serra do Mar”). Sites within this vegetation type were along the coast of Paraná (Antonina) and the Ribeira River Valley (Paraná and São Paulo).
2. Semi-deciduous and Deciduous seasonal forests, in which some tree species lose most or all of their leaves during the dry season, usually the winter in Brazil. These forests are found mainly in the inland and included many of the sites sampled in the States of São Paulo (Taciba, Florínea) and Paraná (Londrina, Campo Mourão, Cornélio Procópio, Jataizinho, Cafeara, Jaguapitã, Sertanópolis, Lerroville);
3. Mixed broadleaf and evergreen forest, with a variable proportion of *Araucariaangustifolia* trees, present particularly in higher elevations of the States of São Paulo, Minas Gerais and Rio de Janeiro and throughout much of the States of Paraná and Santa Catarina, as well as northern Rio Grande do Sul. Sites within this ecoregion were mostly in Paraná (Pinhais, Lapa, Colombo, Curitiba, Ponta Grossa, São Jerônimo da Serra) and Santa Catarina (Canoinhas and Três Barras).

In the Cerrado biome, there are several vegetation types (Oliveira and Marquis 2002), of which the main ones are the grasslands (“dirty” and “clean”, implying areas with a few or no shrubs/small trees), scattered or more dense shrublands (called “Campo Cerrado” and Cerrado “sensu-strictu” in Portuguese) and forested areas with many trees and little (if any) grass undergrowth. Amongst the latter are dense forests like the “Cerradão”, semi-deciduous and deciduous forests, as well as gallery forests along the rivers. Amongst the grasslands are the high-altitude fields with many or few rocks generally on shallow soils (“Campo rupestre”) and the wet fields in lower areas with a high-water table (“Veredas”). These different plant physiognomies show variable grass and canopy cover (and consequently, dominant plant species), fire susceptibility, soil depth and available water. In the present datasets, two of the sites studied were located in ecotone region of the Cerrado with the Atlantic Forest biome (IBGE 2019) in the States of Paraná (Ponta Grossa) and São Paulo (Itatinga). These represent the southernmost portion of this biome (Ritter et al. 2010) and only “clean” grassland and semi-deciduous seasonal forest were sampled in terms of the original vegetation, as well as derived anthropogenic ecosystems like pastures, exotic tree plantations and annual crops.

### Coordinates

-26.27261 and -22.39 Latitude; -53.887222 and -48.470277 Longitude.

## Taxonomic coverage

### Description

A total of 42 taxa were included in the present datasets (Table 1), most of which were arthropods (35 taxa) and, of these, mainly soil or surface-litter dwelling insects (20 taxa). The remainder of the macroinvertebrates collected and reported were Annelids (three taxa), predatory and entomopathogenic nematodes (two taxa), two molluscs (snails and slugs) and the land planarians. Although some of the taxa reported are not typically considered to be soil animals, some of their representatives are frequently associated with the surface litter or the topmost soil layers and, therefore, as they were collected in the samples, they are reported here. These include, for instance, some species of praying-mantis (Mantodea), caddisflies (Trichoptera), lacewings (Chrysopidae) and webspinners (Embioptera). As many larger potworms (Enchytraeida) were found in the samples, these were also collected, though it is well known that the TSBF handsorting method is not efficient at sampling these animals (Niva et al. 2015). Pseudoscorpions, velvet mites and ticks, garden centipedes (Symphyla) and the Diplura, like the potworms, are normally considered as part of the soil mesofauna (Potapov et al. 2022a), but larger individuals are often quite easily visible to the naked eye (Ruiz et al. 2008), so they were included in the current samples. On the other hand, some of the other notable soil fauna representatives were not found in the present samples, for example, the velvet-worms (Onychophora) and solitary bees (Apidae).

## Traits coverage

The collected macroinvertebrate taxa perform various functions in the soil and have a variety of feeding sources, as exemplified in a broad sense in Table 1. Although the datasets do not provide details regarding the traits of the animals collected, information from the Table and from the animal's abundance and biomass can be used to assess potential impacts on soil properties and functions. Ecosystem engineers, like earthworms, termites, ants, millipedes and beetle larvae (Brown et al. 2001), are important ecosystem service providers affecting particularly soil structure and ultimately water availability and storage (Lavelle et al. 2016, Brown et al. 2018). Detritivore soil and litter-dwelling macroinvertebrates, like millipedes, isopods, cockroaches, some beetles, earwigs, fly larvae, booklice and earthworm species, act as major litter decomposers, by directly ingesting leaf-litter or bark or by catalysing microbial colonisation and/or activity (Lavelle 1996, Potapov et al. 2022b). Predators, parasites and microbivores (e.g. bacterivores, fungivores) are important in controlling the populations of other soil organisms, providing pest control services, as well as impacting nutrient cycling (Lavelle 1996, Lavelle et al. 2004). Plant shoot, root or wood-feeding animals affect primary productivity and are often associated with plant pests in agricultural and forestry systems, while parasites, like ticks (Ixodida), affect animal health and welfare (Potapov et al. 2022b).

## Temporal coverage

**Data range:** 2001-2-20 – 2016-9-16.

## Usage licence

### Usage licence

Creative Commons Public Domain Waiver (CC-Zero)

## Data resources

### Data package title

Soil macrofauna biodiversity across land-use systems in Neotropical biomes

### Resource link

https://www.gbif.org/dataset/search?offset=0&publishing_org=bcbe7ef4-5cc8-4197-bccc-1e279fb498a7

### Alternative identifiers

https://collectory.sibbr.gov.br/collectory/public/show/dp76

### Number of data sets

29

### Data set 1.

#### Data set name

Soil macrofauna communities in various land-use systems in Jaguapitã, Paraná, Brazil

#### Data format

Darwin Code Archive

#### Download URL

https://doi.org/10.15468/xjqhra

#### Data format version

1.7

#### Description

Soil macrofauna communities were evaluated in a number of land-use systems on private farms in Jaguapitã, Paraná, Brazil. Sampling was performed in March and September 2004 in nine land uses: 1) a 15-year old pasture (JM) previously sown with *Paspalum* sp. and recently renovated with *Cynodon* sp. grass; 2) a 15-year old degraded pasture of *Paspalum* sp.; 3) a > 15-year old pasture of *Paspalum* sp., recently renovated with Urochloabrizantha; 4) a degraded > 15-year old *Paspalum* sp. pasture; 5) a 6-year old soybean cropping system, established over an old Paspalum sp. pasture; 6) a 2-year old soybean cropping system after long-term *Paspalum* sp. pasture; 7) a recently-renovated *Urochloabrizantha* + *Urochloadecumbens* pasture after several years of annual grain cropping; 8) and 9) two recently-established (1 year old) sugarcane plantations, converted from > 15 year old pastures. In each land use, 25 samples were taken in a square grid of 5 x 5 samples, with 10 m between sampling points. Of the total, five samples (along the diagonal) were taken down to 30 cm depth and the remaining only to 0-10 cm depth. Samples were taken using the standard methodology of the Tropical Soil Biology and Fertility Programme (TSBF), where soil and litter fauna were hand-sorted from monoliths of 25 x 25 cm and the abundance of a total of 42 taxa was assessed.

**Data set 1.** 

Column label	Column description
datasetName	Name of the dataset including information on the biome, locality, county, country and year where the sampling was performed.
basisOfRecord	specific nature of the data collected.
samplingProtocol	method used for sampling the soil macrofauna community.
sampleSizeValue	size of the sample (in square metres).
sampleSizeUnit	unit of sample (in this case m^2^).
eventID	identifier for the broader Event that groups this sampling event.
eventDate	year and month of the sampling, in the format yyyy/month/day.
country	country where sampling occurred.
stateProvince	state where sampling occurred.
county	municipality where sampling occurred, providing the full, unabbreviated name of the smaller administrative region of the sample locality.
locality	full, unabbreviated name of the location where samples were taken.
decimalLatitude	geographic latitude in decimal degrees, using the spatial reference system given in geodeticDatum (WGS84) of the closest known location of the sampling site; when exact coordinates of a particular sample (monolith) was not known, the coordinates are for the overall site (land-use system).
decimalLongitude	geographic longitude in decimal degrees, using the spatial reference system given in geodeticDatum (WGS84) of the closest known location of the sampling site; when exact coordinates of a particular sample (monolith) was not known, the coordinates are for the overall site (land-use system).
habitat	biome according to the Brazilian classification and type of main land-use system; in the present case, samples were taken only in the Atlantic Forest and Cerrado biome, while main land-use systems included mostly agricultural (pastoral, agropastoral, silvopastoral, agrosilvopastoral, annual crops, perennial crops), silvicultural (tree plantations), urban and native vegetation (natural regeneration, forest, grassland).
eventRemarks	season of the sampling event (wet or dry, depending on the precipitation in the month of sampling; wet means more than 100 mm, dry means less than 100 mm rainfall in the month) and Köppen’s climate classification, according to Alvares et al. (2013).
year	year when the sampling event occurred.
month	month of the year when the sampling event occurred.
scientificName	highest level of taxonomic detail provided.
lifeStage	life stage of the invertebrate sampled; in some cases, larvae rather than adults were collected (e.g. for Coleoptera, Diptera and Lepidoptera).
occurrenceID	identifier for the occurrence.
occurrenceStatus	presence/absence of the taxa in the sample.
individualCount	number of individuals sampled (in the individual monolith/sample).
dynamicProperties	includes the relative abundance of each taxon within the sample and the total fresh weight (biomass in grams, if measured) of all the individuals of each taxon weighed together.
kingdom	taxonomic Kingdom.
phyllum	taxonomic Phyllum.
class	taxonomic Class of the invertebrates collected.
order	taxonomic Order of the invertebrates collected.
family	taxonomic Family of the invertebrates collected.
taxonRank	rank of the Taxon provided.
higherClassification	identity of the most detailed taxonomic level provided for the invertebrates collected.
vernacularName	common name of the invertebrate collected.
measurementID	an identifier for the sampling event that includes the particular location (in this case, the individual sample/monolith) where the measurement was made.
measurementType	identifies the particular soil measurement variable evaluated.
measurementValue	individual quantitative value of the particular soil variable measured.
measurementUnit	unit of the variable measured.
measurementMethod	method used to obtain each soil variable measured.

### Data set 2.

#### Data set name

Soil macrofauna communities in various land-use systems in Santo Inácio, Paraná, Brazil

#### Data format

Darwin Core Archive

#### Download URL

https://doi.org/10.15468/dgjpjs

#### Data format version

1.5

#### Description

Soil macrofauna communities were evaluated in a number of land-use systems on a private farm (Estância JAE) in Santo Inácio, Paraná, Brazil. Sampling was performed in October 2013 and January 2014 in seven land uses: 1) an agrosilvopastoral system with *Eucalyptusurograndis* tree rows and *Urochloaruziziensis* pasture and annual crops (soybean, maize, oats, wheat) planted in the inter-row; 2) a silvopastoral system with rows of *Corymbiamaculata* trees and the inter-row planted with *Urochloaruziziensis*; 3) a permanent pasture of *Urochloa* sp.; 4) a sugarcane plantation with conventional tillage; 5) an agropastoral system with soybean in the summer and *Urochloaruziziensis* pasture in the winter; 6) a 20-year old *Eucalyptus* sp. plantation; and 7) a native Atlantic Forest fragment, nearby a stream. Samples were taken using the standard methodology of the Tropical Soil Biology and Fertility Programme (TSBF), where soil (down to 20 cm depth) and litter fauna were hand-sorted from monoliths of 25 x 25 cm and the abundance of a total of 42 taxa was assessed.

**Data set 2.** 

Column label	Column description
idem as Dataset "Soil macrofauna communities in various land-use systems in Jaguapitã, Paraná, Brazil"	idem as Dataset "Soil macrofauna communities in various land-use systems in Jaguapitã, Paraná, Brazil".

### Data set 3.

#### Data set name

Soil macrofauna communities in native Atlantic Forest fragments with different disturbance levels in Londrina, Paraná, Brazil

#### Data format

Darwin Core Archive

#### Download URL

https://doi.org/10.15468/6rxpw9

#### Data format version

1.9

#### Description

Soil macrofauna communities were evaluated in a three semi-deciduous seasonal Atlantic Forest fragments with different levels of disturbance in Londrina, Paraná, Brazil. Sampling was performed in August and December 2005 at the Mata dos Godoy State Park (least disturbed, primary forest), Arthur Thomas Municipal Park (intermediate disturbance; intermediate regeneration stage) and the Horto Florestal of the Universidade Estadual de Londrina (most disturbed; initial regeneration stage). In each forest fragment, 25 samples were taken in a square grid of 5x5 samples, with 10 m between sampling points. Of the total, five samples (along the diagonal) were taken down to 30 cm depth, and the remaining only to 0-10 cm depth. Samples were taken using the standard methodology of the Tropical Soil Biology and Fertility Programme (TSBF), where soil and litter fauna were hand-sorted from monoliths of 25x25 cm, and the abundance of a total of 42 taxa was assessed.

**Data set 3.** 

Column label	Column description
idem as Dataset "Soil macrofauna communities in various land-use systems in Jaguapitã, Paraná, Brazil"	idem as Dataset "Soil macrofauna communities in various land-use systems in Jaguapitã, Paraná, Brazil".

### Data set 4.

#### Data set name

Soil macrofauna communities in various land-use systems in Ponta Grossa, Paraná, Brazil

#### Data format

Darwin Core Archive

#### Download URL

https://doi.org/10.15468/p86sf9

#### Data format version

1.10

#### Description

Soil macrofauna communities were evaluated in in October 2012 and April of 2013 in a number of land-use systems in Ponta Grossa County, Paraná State, Brazil. At the Fazenda Modelo, managed by the Instituto Agronômico do Paraná (IAPAR), samples were taken in three land-use systems as part of a long-term integrated production systems experiment initiated in 2006: 1) an agrosilvopastoral system with rows of *Eucalyptusdunnii* and annual grain cropping (maize, soybean) in the summer and *Loliummultiflorum* (Italian ryegrass) grazed pasture in the winter performed in the inter-row; 2) an agropastoral system with grain cropping (maize, soybean) in the summer and grazed Italian ryegrass in the winter; 3) a permanent pasture of native grasses. Each of these systems was replicated three times and eight samples were taken in each plot. At the Embrapa research Station, two land uses were evaluated, with 24 samples taken in each area: 1) a minimum-tillage grain crop production system; and 2) a 20-year old *Eucalyptusdunnii* tree plantation. Samples were taken using the standard methodology of the Tropical Soil Biology and Fertility Programme (TSBF), where soil (down to 20 cm depth) and litter fauna were hand-sorted from monoliths of 25 x 25 cm and the abundance of a total of 42 taxa was assessed.

**Data set 4.** 

Column label	Column description
idem as Dataset "Soil macrofauna communities in various land-use systems in Jaguapitã, Paraná, Brazil"	idem as Dataset "Soil macrofauna communities in various land-use systems in Jaguapitã, Paraná, Brazil".

### Data set 5.

#### Data set name

Soil macrofauna communities in a pasture and annual cropping systems in Cafeara, Paraná, Brazil

#### Data format

Darwin Core Archive

#### Download URL

https://doi.org/10.15468/wtfx6j

#### Data format version

1.4

#### Description

Soil macrofauna communities were evaluated on six occasions from March 2004 to January 2005 in a pasture and in annual crops following the standard methodology of the Tropical Soil Biology and Fertility Programme (TSBF), in monoliths of 25 x 25 cm, hand-sorted for all litter and soil-dwelling taxa. The abundance of a total of 42 taxa is presented per land use (one pasture and two cropping fields) on each sampling date, performed at a private farm in Cafeara County, Paraná State, Brazil.

**Data set 5.** 

Column label	Column description
idem as Dataset "Soil macrofauna communities in various land-use systems in Jaguapitã, Paraná, Brazil"	idem as Dataset "Soil macrofauna communities in various land-use systems in Jaguapitã, Paraná, Brazil".

### Data set 6.

#### Data set name

Soil macrofauna communities in various land-use systems in Itatinga, São Paulo, Brazil

#### Data format

Darwin Core Archive

#### Download URL

https://doi.org/10.15468/hm49kv

#### Data format version

1.8

#### Description

Soil macrofauna communities were evaluated in February 2014 in a number of land-use systems in Itatinga, São Paulo State, Brazil. At the University of São Paulo Forestry Department Experimental Station, the following land uses were studied: 1) a mixed *Eucalyptusgrandis* and *Acaciamangium* tree plantation; 2) an *Acaciamangium* tree plantation; 3) a *Eucalyptusgrandis* tree plantation; and 4) a Atlantic Forest/Cerrado ecotone semi-deciduous native forest fragment. At a private farm in the Distrito de Lobo, the following land uses were sampled: 1) a mixed *Eucalyptusgrandis* and *Acaciamangium* tree plantation; 2) an *Acaciamangium* tree plantation; 3) a *Eucalyptusgrandis* tree plantation; 4) a sugarcane plantation; and 5) a permanent pasture. At the Fazenda Americana of the Duratex Company, a *Eucalyptus* sp. plantation was sampled on two soil textural types: a sandy clay loam and a clay loam. Samples were taken using the standard methodology of the Tropical Soil Biology and Fertility Programme (TSBF), where soil (down to 20 cm depth) and litter fauna were hand-sorted from monoliths of 25 x 25 cm and the abundance of a total of 42 taxa was assessed.

**Data set 6.** 

Column label	Column description
idem as Dataset "Soil macrofauna communities in various land-use systems in Jaguapitã, Paraná, Brazil"	idem as Dataset "Soil macrofauna communities in various land-use systems in Jaguapitã, Paraná, Brazil".

### Data set 7.

#### Data set name

Soil macrofauna communities in urban public parks of Curitiba, Paraná State, Brazil

#### Data format

Darwin Core Archive

#### Download URL

https://doi.org/10.15468/ek2wuh

#### Data format version

1.7

#### Description

Soil macrofauna communities were evaluated in five urban parks of the City of Curitiba, Paraná State, Brazil: Barigui, Tingui, Barreirinha and Passaúna Municipal parks and the Botanic Garden. Samples were taken in November 2013 and July 2014 in two areas of each park: one with native Atlantic Forest and the other with a grass lawn. Five samples were taken in each land use on each date, using the standard methodology of the Tropical Soil Biology and Fertility Programme (TSBF), where soil (down to 20 cm depth) and litter fauna were hand-sorted from monoliths of 25 x 25 cm and the abundance of a total of 42 taxa was assessed.

**Data set 7.** 

Column label	Column description
idem as Dataset "Soil macrofauna communities in various land-use systems in Jaguapitã, Paraná, Brazil"	idem as Dataset "Soil macrofauna communities in various land-use systems in Jaguapitã, Paraná, Brazil".

### Data set 8.

#### Data set name

Soil macrofauna communities in a long-term soil and crop management experiment at Embrapa Soybean, Londrina, Paraná, Brazil

#### Data format

Darwin Core Archive

#### Download URL

https://doi.org/10.15468/3gbqk9

#### Data format version

1.5

#### Description

The soil macrofauna community was assessed in a long-term soil and crop management experiment established in 1988 at the Embrapa Soybean Research Station in Londrina, Paraná, Brazil. The experiment includes two rotation systems (soybean-wheat double-cropping and a rotation with lupine/maize-oats/soybean-wheat/soybean-wheat/soybean) and three soil tillage types (no-tillage, conventional tillage and minimum tillage with chisel plough every 3 years). The experiment was replicated four times and one sample was taken in each plot. Sampling was performed in April 2001 and April 2005, using the standard methodology of the Tropical Soil Biology and Fertility Programme (TSBF). Monoliths were hand-sorted for all litter and soil-dwelling taxa (down to 30 cm depth) and the abundance of a total of 42 taxa assessed.

**Data set 8.** 

Column label	Column description
idem as Dataset "Soil macrofauna communities in various land-use systems in Jaguapitã, Paraná, Brazil"	idem as Dataset "Soil macrofauna communities in various land-use systems in Jaguapitã, Paraná, Brazil".

### Data set 9.

#### Data set name

Soil macrofauna communities in an organic agroforestry system and under initial native vegetation regeneration at the Assentamento Contestado in Lapa, Paraná, Brazil

#### Data format

Darwin Core Archive

#### Download URL

https://doi.org/10.15468/rmfcj7

#### Data format version

1.4

#### Description

Soil macrofauna communities were evaluated in an organic agroforestry production system and in initial native vegetation regeneration at an area managed and owned by the Assentamento Contestado, in Lapa, Paraná, Brazil. Sampling was performed in April 2016 in three areas with agroforestry systems including various vegetables, pasture grasses and orchard trees, like figs, peaches, pears, apples, pecans and persimmons. Samples were taken using the standard methodology of the Tropical Soil Biology and Fertility Programme (TSBF), where soil (down to 20 cm depth) and litter fauna were hand-sorted from monoliths of 25 x 25 cm and the abundance of a total of 42 taxa was assessed.

**Data set 9.** 

Column label	Column description
idem as Dataset "Soil macrofauna communities in various land-use systems in Jaguapitã, Paraná, Brazil"	idem as Dataset "Soil macrofauna communities in various land-use systems in Jaguapitã, Paraná, Brazil".

### Data set 10.

#### Data set name

Soil macrofauna communities in native Atlantic Forest fragments and agroforestry systems of different ages the Ribeira River Valley, Brazil

#### Data format

Darwin Core Archive

#### Download URL

https://doi.org/10.15468/h6m947

#### Data format version

1.7

#### Description

Soil macrofauna communities were evaluated in three Atlantic Forest fragments at different stages of regeneration and in three organic agroforestry systems of different ages in the Ribeira River Valley, Brazil. Sampling was performed in March and August of 2008 in six sites, five of which were located in Barra do Turvo County in São Paulo State and one in Adrianópolis County in Paraná State. The sites in Barra do Turvo were: a 5, 20 and > 30-year-old Atlantic Forest regeneration fragment and a 4- and 16-year-old organic agroforestry system. In Adrianópolis, only an 8-year-old organic agroforestry system was sampled. Each land use was replicated three times and two samples were taken per plot. Samples were taken using the standard methodology of the Tropical Soil Biology and Fertility Programme (TSBF), where soil (down to 20 cm depth) and litter fauna were hand-sorted from monoliths of 25 x 25 cm and the abundance of a total of 42 taxa was assessed.

**Data set 10.** 

Column label	Column description
idem as Dataset "Soil macrofauna communities in various land-use systems in Jaguapitã, Paraná, Brazil"	idem as Dataset "Soil macrofauna communities in various land-use systems in Jaguapitã, Paraná, Brazil".

### Data set 11.

#### Data set name

Soil macrofauna communities in various land uses in and neighbouring the Mata do Uru Private Reserve, Lapa, Paraná State, Brazil

#### Data format

Darwin Core Archive

#### Download URL

https://doi.org/10.15468/h8y7f5

#### Data format version

1.7

#### Description

Soil macrofauna communities were evaluated in four land uses in the Mata do Uru Private Reserve and in a conventionally managed annual grain crop production system neighbouring the Reserve, in February 2015 and July 2016. In the Uru Reserve, samples were taken in: 1) a native Atlantic Forest (*Araucaria* forest) fragment; 2) a 10-year-old regeneration plot, planted with native trees; 3) a native grassland; and 4) a grass lawn. Six samples were taken in each land use on each date, using the standard methodology of the Tropical Soil Biology and Fertility Programme (TSBF), where soil (down to 20 cm depth) and litter fauna were hand-sorted from monoliths of 25 x 25 cm and the abundance of a total of 42 taxa was assessed.

**Data set 11.** 

Column label	Column description
idem as Dataset "Soil macrofauna communities in various land-use systems in Jaguapitã, Paraná, Brazil"	idem as Dataset "Soil macrofauna communities in various land-use systems in Jaguapitã, Paraná, Brazil".

### Data set 12.

#### Data set name

Soil macrofauna communities in native Atlantic Forest and native grassland vegetation at the Vila Velha State Park, Ponta Grossa, Paraná, Brazil

#### Data format

Darwin Core Archive

#### Download URL

https://doi.org/10.15468/9ja9ce

#### Data format version

1.6

#### Description

Soil macrofauna communities were evaluated in a native Atlantic Forest (*Araucaria* forest) and a native grassland area at the Vila Velha State Park, in Ponta Grossa County, Paraná State, Brazil, in September 2013 and in January 2014. The fauna was sampled following the standard methodology of the Tropical Soil Biology and Fertility Programme (TSBF), where the soil (down to 20 cm depth) and surface litter were hand-sorted from monoliths of 25 x 25 cm and the abundance of a total of 42 taxa assessed.

**Data set 12.** 

Column label	Column description
idem as Dataset "Soil macrofauna communities in various land-use systems in Jaguapitã, Paraná, Brazil"	idem as Dataset "Soil macrofauna communities in various land-use systems in Jaguapitã, Paraná, Brazil".

### Data set 13.

#### Data set name

Soil macrofauna communities in a sandy soil with conventional and no-tillage soybean production systems in Taciba, São Paulo State, Brazil

#### Data format

Darwin Core Archive

#### Download URL

https://doi.org/10.15468/hfpvrg

#### Data format version

1.5

#### Description

Soil macrofauna communities were evaluated in a private farm with a soybean production system using conventional tillage and no-tillage after long-term permanent pastures in Taciba, São Paulo State, Brazil. Samples were taken in December of 2004 in two areas with no-tillage (second and third year of no-tillage soybean production) and in a recently-tilled (conventional) soybean production area converted from pasture. Four samples were taken in each area, using the standard methodology of the Tropical Soil Biology and Fertility Programme (TSBF), where soil (down to 30 cm depth) and litter fauna were hand-sorted from monoliths of 25 x 25 cm and the abundance of a total of 42 taxa was assessed.

**Data set 13.** 

Column label	Column description
idem as Dataset "Soil macrofauna communities in various land-use systems in Jaguapitã, Paraná, Brazil"	idem as Dataset "Soil macrofauna communities in various land-use systems in Jaguapitã, Paraná, Brazil"

### Data set 14.

#### Data set name

Soil macrofauna communities in various land-use systems in Canoinhas and Três Barras, Santa Catarina State, Brazil

#### Data format

Darwin Core Archive

#### Download URL

https://doi.org/10.15468/2r7dpw

#### Data format version

1.7

#### Description

Soil macrofauna communities were evaluated in May 2011 in five land-use systems in the regions of Canoinhas and Três Barras, Santa Catarina State, Brazil. In Três Barras, samples were taken in a native Atlantic Forest fragment and an *Araucariaangustifolia* tree plantation in the Três Barras National Forest, as well as in an annual grain crop production system under no-tillage on a private farm. In Canoinhas, samples were taken in an agropastoral system with Italian ryegrass (*Loliummultiflorum*) and in a permanent pasture of native grasses. Nine samples were taken in each land-use system in a square grid with 30 m distance between samples. Samples were taken using the standard methodology of the Tropical Soil Biology and Fertility Programme (TSBF), where soil (down to 20 cm depth) and litter fauna were hand-sorted from monoliths of 25 x 25 cm and the abundance of a total of 42 taxa was assessed.

**Data set 14.** 

Column label	Column description
idem as Dataset "Soil macrofauna communities in various land-use systems in Jaguapitã, Paraná, Brazil"	idem as Dataset "Soil macrofauna communities in various land-use systems in Jaguapitã, Paraná, Brazil".

### Data set 15.

#### Data set name

Soil macrofauna communities in an integrated production system experiment at the Canguiri Farm in Pinhais, Paraná, Brazil

#### Data format

Darwin Core Archive

#### Download URL

https://doi.org/10.15468/5x7dwu

#### Data format version

1.9

#### Description

Soil macrofauna communities were evaluated in September 2016 in an experiment evaluating integrated agricultural production systems, located at the Canguiri Farm of the Federal University of Paraná in Pinhais, Paraná, Brazil. Samples were taken in four land-use systems: 1) an agrosilvopastoral system with rows of *Eucalyptusbenthamii* and maize cropping in the summer and black oats in the winter, followed by three years of *Panicummaximum* pasture grasses in the inter-row; 2) an agropastoral system with maize cropping in the summer and black oats in the winter, followed by three years of *Panicummaximum* pasture grasses in the inter-row; 3) a permanent pasture of *Panicummaximum*; and 4) annual cropping of maize in the summer and black oats in the winter. Each of these systems was replicated three times and three samples were taken in each plot. Samples were taken using the standard methodology of the Tropical Soil Biology and Fertility Programme (TSBF), where soil (down to 20 cm depth) and litter fauna were hand-sorted from monoliths of 25 x 25 cm and the abundance of a total of 42 taxa was assessed.

**Data set 15.** 

Column label	Column description
idem as Dataset "Soil macrofauna communities in various land-use systems in Jaguapitã, Paraná, Brazil"	idem as Dataset "Soil macrofauna communities in various land-use systems in Jaguapitã, Paraná, Brazil"

### Data set 16.

#### Data set name

Soil macrofauna communities in native tree plantations along the coastal plain of the State of Paraná, Antonina, Brazil

#### Data format

Darwin Core Archive

#### Download URL

https://doi.org/10.15468/xagmsg

#### Data format version

1.6

#### Description

Soil macrofauna communities were evaluated in two native tree plantations used to recover native vegetation in abandoned pastures on the coastal plain of the State of Paraná, Brazil. Samples were taken in November 2007 in the Cachoeira River Natural Reserve managed by the “Sociedade de Proteção da Vida Silvestre” (SPVS) in Antonina County. Samples were taken in *Ingaedulis* and *Myrsinecoreacea* plantations, replicated four times. Two samples were taken in each plot using the standard methodology of the Tropical Soil Biology and Fertility Programme (TSBF), where soil (taken only to 10 cm depth due to the high water table) and litter fauna were hand-sorted from monoliths of 25 x 25 cm and the abundance of a total of 42 taxa was assessed.

**Data set 16.** 

Column label	Column description
idem as Dataset "Soil macrofauna communities in various land-use systems in Jaguapitã, Paraná, Brazil"	idem as Dataset "Soil macrofauna communities in various land-use systems in Jaguapitã, Paraná, Brazil".

### Data set 17.

#### Data set name

Soil macrofauna communities in a long-term soil tillage experiment at Embrapa Soybean, Londrina, Paraná, Brazil

#### Data format

Darwin Core Archive

#### Download URL

https://doi.org/10.15468/pucqj2

#### Data format version

1.4

#### Description

The soil macrofauna community was assessed in a long-term soil tillage experiment established in 1981 at the Embrapa Soybean Research Station in Londrina, Paraná, Brazil. The experiment is conducted under soybean-wheat double-cropping and using three soil tillage types: no-tillage, conventional tillage (disc plough) and minimum tillage (chisel plough every 3 years). The experiment was replicated four times and one sample was taken in each plot. Sampling was performed in September 2001 and October 2005, using the standard methodology of the Tropical Soil Biology and Fertility Programme (TSBF). Monoliths were hand-sorted for all litter and soil-dwelling taxa (down to 30 cm depth) and the abundance of a total of 42 taxa was assessed.

**Data set 17.** 

Column label	Column description
idem as Dataset "Soil macrofauna communities in various land-use systems in Jaguapitã, Paraná, Brazil"	idem as Dataset "Soil macrofauna communities in various land-use systems in Jaguapitã, Paraná, Brazil".

### Data set 18.

#### Data set name

Soil macrofauna communities in a long-term crop rotation experiment at Coamo in Campo Mourão, Paraná, Brazil

#### Data format

Darwin Core Archive

#### Download URL

https://doi.org/10.15468/gxz6q2

#### Data format version

1.4

#### Description

The soil macrofauna community was assessed in a long-term crop rotation experiment established in 1985 at the Coamo Agroindustrial Cooperativa experimental farm in Campo Mourão, Paraná State, Brazil. The experiment includes several rotations, all of which are planted using the no-tillage system, though only four rotation systems were evaluated: maize-oats/soybean-maize-millet/soybean-maize/soybean-wheat (Tr3), maize-lupine/soybean-oats/soybean-wheat/soybean-wheat (Tr4), maize-hairy vetch/maize-maize-millet/soybean-maize/soybean-wheat (Tr8) and soybean-wheat continuous double-cropping (Tr11). The experiment was replicated four times and one sample was taken in each plot. Sampling was performed in April 2004, using the standard methodology of the Tropical Soil Biology and Fertility Programme (TSBF). Monoliths were hand-sorted for all litter and soil-dwelling taxa (down to 30 cm depth) and the abundance of a total of 42 taxa was assessed.

**Data set 18.** 

Column label	Column description
idem as Dataset "Soil macrofauna communities in various land-use systems in Jaguapitã, Paraná, Brazil"	idem as Dataset "Soil macrofauna communities in various land-use systems in Jaguapitã, Paraná, Brazil".

### Data set 19.

#### Data set name

Soil macrofauna communities in an organic grain production system under conventional and no-tillage systems in São Jerônimo da Serra, Paraná, Brazil

#### Data format

Darwin Core Archive

#### Download URL

https://doi.org/10.15468/fxgq4p

#### Data format version

1.4

#### Description

Soil macrofauna communities were evaluated in an organic grain (soybean) crop production system at the farm run by the Associação Filantrópica Humanitas in São Jerônimo da Serra, Paraná, Brazil. Sampling was performed in April 2003 in an area planted with soybean under conventional tillage and another area planted with no-tillage system for two years. Samples were taken using the standard methodology of the Tropical Soil Biology and Fertility Programme (TSBF), where soil (down to 30 cm depth) and litter fauna were hand-sorted from monoliths of 25 x 25 cm and the abundance of a total of 42 taxa was assessed.

**Data set 19.** 

Column label	Column description
idem as Dataset "Soil macrofauna communities in various land-use systems in Jaguapitã, Paraná, Brazil"	idem as Dataset "Soil macrofauna communities in various land-use systems in Jaguapitã, Paraná, Brazil".

### Data set 20.

#### Data set name

Soil macrofauna communities in organic grain production systems in Jataizinho, Paraná, Brazil

#### Data format

Darwin Core Archive

#### Download URL

https://doi.org/10.15468/dfdh2j

#### Data format version

1.6

#### Description

Soil macrofauna communities were evaluated in two annual grain crop (soybean and maize) organic production systems in the Municipality of Jataizinho, Paraná, Brazil, in April 2003. Sampling followed the standard methodology of the Tropical Soil Biology and Fertility Programme (TSBF), where soil (down to 40 cm depth) and litter fauna were hand-sorted from monoliths of 25 x 25 cm and the abundance of a total of 42 taxa was assessed.

**Data set 20.** 

Column label	Column description
idem as Dataset "Soil macrofauna communities in various land-use systems in Jaguapitã, Paraná, Brazil"	idem as Dataset "Soil macrofauna communities in various land-use systems in Jaguapitã, Paraná, Brazil".

### Data set 21.

#### Data set name

Soil macrofauna communities in a long-term soil tillage experiment at Coamo in Campo Mourão, Paraná, Brazil

#### Data format

Darwin Core Archive

#### Download URL

https://doi.org/10.15468/595jyb

#### Data format version

1.6

#### Description

The soil macrofauna community was assessed in a long-term soil tillage systems experiment established in 1991 at the Coamo Agroindustrial Cooperativa experimental farm in Campo Mourão, Paraná State, Brazil. The experiment includes three soil tillage systems (no-tillage, conventional tillage and minimum tillage) planted with two crop rotation systems: soybean-wheat continuous double-cropping and a more complex rotation (including maize, wheat, oats, soybean and lupine). However, only four treatments were evaluated: no-tillage with complex rotation; no-tillage with double-cropping; conventional (disc) tillage with double-cropping, minimum tillage (chisel ploughing every 3 years) with double-cropping. Four samples were taken per treatment in April 2004, using the standard methodology of the Tropical Soil Biology and Fertility Programme (TSBF). Monoliths were hand-sorted for all litter and soil-dwelling taxa (down to 30 cm depth) and the abundance of a total of 42 taxa assessed.

**Data set 21.** 

Column label	Column description
idem as Dataset "Soil macrofauna communities in various land-use systems in Jaguapitã, Paraná, Brazil"	idem as Dataset "Soil macrofauna communities in various land-use systems in Jaguapitã, Paraná, Brazil".

### Data set 22.

#### Data set name

Soil macrofauna communities along a transect from native Atlantic Forest at the Mata dos Godoy State Park (Londrina, Paraná, Brazil) into a soybean cultivation field

#### Data format

Darwin Core Archive

#### Download URL

https://doi.org/10.15468/9ze9v2

#### Data format version

1.7

#### Description

The soil macrofauna community was assessed along a transect including 16 sampling points from the inside of the forest (80 m from the edge) out towards the neighbouring recently-harvested soybean plantation (up to 70 m from the edge). Sampling was performed in May 2003 at the Mata dos Godoy State Park and the neighbouring Fazenda Santa Helena, using the standard methodology of the Tropical Soil Biology and Fertility Programme (TSBF). Monoliths were hand-sorted for all litter and soil-dwelling taxa (down to a 40 cm depth) and the abundance of a total of 42 taxa was assessed.

**Data set 22.** 

Column label	Column description
idem as Dataset "Soil macrofauna communities in various land-use systems in Jaguapitã, Paraná, Brazil"	idem as Dataset "Soil macrofauna communities in various land-use systems in Jaguapitã, Paraná, Brazil".

### Data set 23.

#### Data set name

Soil macrofauna communities in native Atlantic Forest fragments in Sertanópolis, Paraná, Brazil

#### Data format

Darwin Core Archive

#### Download URL

https://doi.org/10.15468/6r73ze

#### Data format version

1.8

#### Description

Soil macrofauna communities were evaluated in two native Atlantic Forest fragments in private properties in the Municipality of Sertanópolis, in northern Paraná State, Brazil, in October 2001 and in April 2003. Sampling followed the standard methodology of the Tropical Soil Biology and Fertility Programme (TSBF), where soil (down to 40 cm depth) and litter fauna were hand-sorted from monoliths of 25 x 25 cm and the abundance of a total of 42 taxa was assessed.

**Data set 23.** 

Column label	Column description
idem as Dataset "Soil macrofauna communities in various land-use systems in Jaguapitã, Paraná, Brazil"	idem as Dataset "Soil macrofauna communities in various land-use systems in Jaguapitã, Paraná, Brazil".

### Data set 24.

#### Data set name

Soil macrofauna communities along a transect from native Atlantic Forest into a soybean cultivation field at the Fazenda São Paulo in Cornélio Procópio, Paraná, Brazil

#### Data format

Darwin Core Archive

#### Download URL

https://doi.org/10.15468/qfzdte

#### Data format version

1.4

#### Description

The soil macrofauna community was assessed along a transect including 13 sampling points from the inside of the forest (60 m from the edge) out towards the neighbouring recently-harvested soybean plantation (up to 60 m from the edge). Sampling was performed in April 2004 at the Fazenda São Paulo, near Cornélio Procópio, Paraná State, Brazil, using the standard methodology of the Tropical Soil Biology and Fertility Programme (TSBF). Monoliths were hand-sorted for all litter and soil-dwelling taxa (down to 30 cm depth) and the abundance of a total of 42 taxa was assessed.

**Data set 24.** 

Column label	Column description
idem as Dataset "Soil macrofauna communities in various land-use systems in Jaguapitã, Paraná, Brazil"	idem as Dataset "Soil macrofauna communities in various land-use systems in Jaguapitã, Paraná, Brazil".

### Data set 25.

#### Data set name

Soil macrofauna community in areas with high and low population of Scarab beetle larvae in Lerroville, Paraná, Brazil

#### Data format

Darwin Core Archive

#### Download URL

https://doi.org/10.15468/hcj7af

#### Data format version

1.7

#### Description

The soil macrofauna community was assessed in a long-term (28 years) no-tillage annual grain production farm with areas having a high and a low population of Scarab beetle larvae in Lerroville, Londrina Municipality, Paraná State, Brazil. Sampling occurred after the soybean harvest in April 2003, using the standard methodology of the Tropical Soil Biology and Fertility Programme (TSBF). Monoliths were hand-sorted for all litter and soil-dwelling taxa (down to 30 cm depth) and the abundance of a total of 42 taxa was assessed.

**Data set 25.** 

Column label	Column description
idem as Dataset "Soil macrofauna communities in various land-use systems in Jaguapitã, Paraná, Brazil"	idem as Dataset "Soil macrofauna communities in various land-use systems in Jaguapitã, Paraná, Brazil".

### Data set 26.

#### Data set name

Soil macrofauna communities in an early conversion phase of conventional to organic grain production systems at the Embrapa Soybean Experiment Station in Londrina, Paraná, Brazil

#### Data format

Darwin Core Archive

#### Download URL

https://doi.org/10.15468/hya4u8

#### Data format version

1.5

#### Description

Soil macrofauna communities were evaluated in two areas recently converted from conventional to organic grain (soybean) crop production systems at the Embrapa Soybean Experiment Station in Londrina, Paraná, Brazil. Sampling was performed in October 2003 in an area planted with soybean under conventional tillage and an area planted with pigeon-pea (*Cajanuscajan*) under a no-tillage system. Samples were taken using the standard methodology of the Tropical Soil Biology and Fertility Programme (TSBF), where soil (down to 30 cm depth) and litter fauna were hand-sorted from monoliths of 25 x 25 cm and the abundance of a total of 42 taxa was assessed.

**Data set 26.** 

Column label	Column description
idem as Dataset "Soil macrofauna communities in various land-use systems in Jaguapitã, Paraná, Brazil"	idem as Dataset "Soil macrofauna communities in various land-use systems in Jaguapitã, Paraná, Brazil".

### Data set 27.

#### Data set name

Soil macrofauna communities in native Atlantic Forest fragments and *Pinus* plantations at the Embrapa Forestry Research Station in Colombo, Paraná, Brazil

#### Data format

Darwin Core Archive

#### Download URL

https://doi.org/10.15468/w6v87y

#### Data format version

1.5

#### Description

Soil macrofauna communities were evaluated in four native Atlantic Forest (*Araucaria* forests) fragments and three *Pinuselliottii* plantations at the Embrapa Forestry Research Station in Colombo Municipality, Paraná, Brazil. Three forest fragments in an advanced state of regeneration (> 70 years without disturbance) and three pine plantations (25 to 32 years old) were sampled in May 2007 and another forest fragment was sampled in September 2011, following the standard methodology of the Tropical Soil Biology and Fertility Programme (TSBF). Soil and litter fauna were hand-sorted from monoliths of 25 x 25 cm (to 10 cm depth in 2007 and 20 cm depth in 2011) and the abundance of a total of 42 taxa was assessed.

**Data set 27.** 

Column label	Column description
idem as Dataset "Soil macrofauna communities in various land-use systems in Jaguapitã, Paraná, Brazil"	idem as Dataset "Soil macrofauna communities in various land-use systems in Jaguapitã, Paraná, Brazil".

### Data set 28.

#### Data set name

Soil macrofauna communities in native Atlantic Forest at the Mata dos Godoy State Park in Londrina, Paraná, Brazil

#### Data format

Darwin Core Archive

#### Download URL

https://doi.org/10.15468/43j5fq

#### Data format version

1.4

#### Description

Soil macrofauna communities were evaluated in February 2001 in a native fragment forest at the Mata dos Godoy State Park using the standard methodology of the Tropical Soil Biology and Fertility Programme (TSBF). Monoliths were hand-sorted for all litter and soil-dwelling taxa (down to a 40 cm depth) and the abundance of a total of 42 taxa was assessed.

**Data set 28.** 

Column label	Column description
idem as Dataset "Soil macrofauna communities in various land-use systems in Jaguapitã, Paraná, Brazil"	idem as Dataset "Soil macrofauna communities in various land-use systems in Jaguapitã, Paraná, Brazil".

### Data set 29.

#### Data set name

Soil macrofauna community in a site with high and low population of Scarab beetle larvae in Florínea, São Paulo, Brazil

#### Data format

Darwin Core Archive

#### Download URL

https://doi.org/10.15468/6byztf

#### Data format version

1.8

#### Description

The soil macrofauna community was assessed in a no-tillage annual grain production farm with areas having a high and low population of Scarab beetle larvae in Florínea, São Paulo State, Brazil. The site had been in maize production and the sampling occurred after the maize harvest in March 2005, using the standard methodology of the Tropical Soil Biology and Fertility Programme (TSBF). Monoliths were hand-sorted for all litter and soil-dwelling taxa (down to 40 cm depth) and the abundance of a total of 42 taxa was assessed.

**Data set 29.** 

Column label	Column description
idem as Dataset "Soil macrofauna communities in various land-use systems in Jaguapitã, Paraná, Brazil"	idem as Dataset "Soil macrofauna communities in various land-use systems in Jaguapitã, Paraná, Brazil".

## Additional information

The 29 datasets are described individually above, including a title and a brief summary (as provided in the IPT website of Embrapa Forestry; see https://www.gbif.org/publisher/bcbe7ef4-5cc8-4197-bccc-1e279fb498a7) of each study, while the overall information on number of sites, plots or land-use systems sampled, as well as the number of records (monoliths) and occurrences and soil metadata measures are listed in Table 2. All datasets include the abundance and (frequently) biomass of the 42 soil macroinvertebrate taxa listed in Table 1. Köppen Climate types provided followed Alvares et al. (2013). Furthermore, information on variables included in the "Extended measurement or fact" datasheets (soil chemical and physical properties) is shown in Table 3. The number of soil variables in each dataset depended on the intensity of measurements performed at each particular site, but generally included the standard chemical attributes analysed in Brazilian soil analysis laboratories, i.e. pH, Exchangeable Potassium, Calcium, Magnesium and Aluminium, Potential acidity, Available Phosphorus, Soil Organic Carbon, Sum of bases, Cation Exchange Capacity and Base saturation. Infrequently, soil organic nitrogen was measured. The vast majority of the sites also had particle size distribution results (i.e. sand, silt and clay contents), while only a few had data on bulk density and soil moisture.

## Supplementary Material

B4B2DB1C-9FEA-5F92-8EEF-95D5433D455C 10.3897/BDJ.12.e115000.suppl1 Supplementary material 1 Table showing additional sites for which soil macrofauna data are available in Brazil Data type PDF Brief description Table showing additional sites for which soil macrofauna data are available in Brazil. These data were provided by a large number of authors/collaborators and were prepared for the FaunaServices project using the standard template of Mathieu et al. (2023a). Additional sites (Counties and Brazilian States identified with standard abbreviations) and land-use systems in various Brazilian biomes (IBGE, 2019) where soil macroinvertebrate communities were sampled using the standard TSBF method in Brazil and for which data are available at the monolith and/or layer level for up to 42 taxa. The number of sites, plots or treatments sampled and the number of records, occurrences and soil-related data in each dataset are also provided. File: oo_962094.pdf https://binary.pensoft.net/file/962094 Wilian C. Demetrio, Quentin Gabriac, George G. Brown

## Figures and Tables

**Table 1.** List of the 42 taxa of soil macroinvertebrates considered in the current datasets (including common names, when present) and an indication of their main food preferences and functional groups. Updated from Brown et al. (2018) and complemented with information from Potapov et al. (2022b).

**Taxonomic classification**	**Common name**	**Feeding preferences & Functional groups**				
		**Geophage, Bioturbator**	**Detritivore, Coprophage, Decomposer**	**Phytophage, Xylophage, Pest**	**Carnivore, Predator, Parasite**	**Fungivore, Microbivore**
**Phylum Annelida**						
Class Clitellata						
Subclass Hirudinea	Leeches				X	
Subclass Oligochaeta						
Order Crassiclitellata	Earthworms	X	X			X
Order Enchytraeida	Enchytraeids, potworms	X	X			X
**Phylum Mollusca**						
Class Gastropoda	Slugs and snails	X	X	X		X
**Phylum Nematoda**						
Class Enoplea						
Order Mermithida	Mermithid				X	
Class Gordioidea						
Order Gordioida	Horsehair worms				X	
**Phylum Platyhelminthes**						
Class Rhabditophora						
Order Tricladida	Flatworms, land planarians				X	
**Phylum Arthropoda**						
Subphylum Chelicerata						
Class Arachnida						
Order Trombidiformes	Velvet mites				X	
Order Amblypigi	Whip-spiders					
Order Araneae	Spiders	X			X	
Order Ixodida	Ticks				X	
Order Opiliones	Harvestmen	X			X	
Order Pseudoscorpiones	Pseudoscorpions		X		X	X
Order Scorpiones	Scorpions	X			X	
Order Solifugae	Camel spiders	X			X	
Order Uropygi	Vinagroon scorpions				X	
Subphylum Crustacea						
Class Malacostraca						
Order Amphipoda						
Family Talitridae	Sandfleas	X	X			
Order Isopoda						
Suborder Oniscidea	Woodlice, pillbugs, sowbugs	X	X		X	X
Subphylum Hexapoda						
Class Diplura	ND		X		X	
Class Insecta						
Order Achaeognatha	Bristletails		X	X		
Order Blattodea: Blattaria	Cockroaches	X	X	X		X
Isoptera	Termites	X	X	X		X
Order Coleoptera	Beetles	X	X	X	X	X
Order Dermaptera	Earwigs	X	X	X	X	
Order Diptera	Fly larvae	X	X	X	X	X
Order Embioptera	Webspinners		X			X
Order Hemiptera		X	X	X	X	
Suborder Auchenorrhyncha	Cicadas	X		X		
Suborder Heteroptera	True bugs	X		X	X	
Order Hymenoptera						
Family Formicidae	Ants	X	X	X	X	X
Family Vespidae	Wasps, hornets	X			X	
Order Lepidoptera	Butterflies, moths (larvae, pupae)			X	X	
Order Mantodea	Praying mantis				X	
Order Neuroptera						
Family Myrmeleontidae	Antlions	X			X	
Family Chrysopidae	Lacewings				X	
Order Orthoptera	Crickets	X		X		
Order Psocodea	Booklice		X	X		
Order Thysanoptera	Thrips		X	X	X	
Order Trichoptera	Caddisflies		X		X	X
Order Zygentoma	Silverfish		X			X
Subphylum Myriapoda						
Class Chilopoda	Centipedes	X			X	
Class Diplopoda	Millipedes	X	X	X		X
Class Symphyla	Garden centipedes		X	X	X	X

**Table 2.** Sites (Counties and States following the Brazilian abbreviation, where PR = Paraná, SC = Santa Catarina and SP = São Paulo), approximate geographic location land-use systems and number of plots or treatments evaluated and the number of records, occurrences and metadata including various soil attributes of the 29 datasets on soil macrofauna communities in Brazil made available online (published) in the GBIF system via the Embrapa Forestry IPT (see https://www.gbif.org/dataset/search?offset=0&publishing_org=bcbe7ef4-5cc8-4197-bccc-1e279fb498a7), as part of the project “Soil macrofauna communities in Brazilian land-use systems“, through a SoilBON/CSU-GBIF data mobilisation call (Guerra and Wall 2023).

**Counties (State)**	**Latitude**	**Longitude**	**Land use systems**	**No. sites, plots, treatments**	**Link to published dataset in GBIF**	**Records**	**Occurrence**	**Soil**	**Reference**
Jaguapitã (PR)	-23.04722	-51.54555	native vegetation / pasture / annual crop	9	https://doi.org/10.15468/xjqhra	450	20,250	7,649	Benito et al. 2023
Itatinga (SP)	-23.04251	-48.63158	native vegetation / annual crop / pasture / forestry plantation	11	https://doi.org/10.15468/hm49kv	144	6,480	2,160	Cremonesi et al. 2023
Santo Inácio (PR)	-22.76638	-51.85083	perennial crop / pasture / agropastoral and agrosilvopastoral systems / forestry plantation / native vegetation	7	https://doi.org/10.15468/dgjpjs	156	7,020	1,716	Nadolny et al. 2023b
Ponta Grossa (PR)	-25.08638	-50.16055	Agrosilvopastoral and agropastoral system / forestry plantation / perennial crop / pasture	5	https://doi.org/10.15468/p86sf9	150	6,450	2,550	Zagatto et al. 2023
Curitiba (PR)	-25.4257	-49.31163	native vegetation / urban lawn	10	https://doi.org/10.15468/ek2wuh	100	4,500	1,700	Bartz et al. 2023
Londrina (PR)	-23.44527	-51.24944	native vegetation	3	https://doi.org/10.15468/6rxpw9	150	6,750	2,550	Korasaki et al. 2023c
Cafeara (PR)	-22.83575	-51.70034	annual crop / pasture	3	https://doi.org/10.15468/wtfx6j	86	3,870	492	Korasaki et al. 2023b
Adrianópolis (PR), Barra do Turvo (SP)	-24.88666	-48.48194	agroforestry systems / native vegetation	6	https://doi.org/10.15468/h6m947	72	3,240	1,224	Maschio et al. 2023
Ponta Grossa (PR)	-25.23952	-49.9985	native vegetation	2	https://doi.org/10.15468/9ja9ce	60	2,700	480	Santos et al. 2023
Lapa (PR)	-25.80932	-49.68626	annual crop / native vegetation	5	https://doi.org/10.15468/h8y7f5	60	2,700	1,020	Dudas et al. 2023
Londrina (PR)	-23.18527	-51.17833	annual crop	8	https://doi.org/10.15468/3gbqk9	56	2,520	336	Brown et al. 2023m
Lapa (PR)	-25.65192	-49.70067	agroforestry / native vegetation	5	https://doi.org/10.15468/rmfcj7	41	1,800	336	Nadolny et al. 2023c
Três Barras, Canoinhas (SC)	-26.18804	-50.22586	annual crop / forestry plantation / agropastoral system / native vegetation / pasture	5	https://doi.org/10.15468/2r7dpw	45	2,025	765	Clasen et al. 2023
Pinhais (PR)	-25.40172	-49.1225	annual crop / pasture / agropastoral and agrosilvopastoral system	4	https://doi.org/10.15468/5x7dwu	36	1,620	612	Ferreira et al. 2023
Antonina (PR)	-25.41666	-48.66666	Native tree plantation	2	https://doi.org/10.15468/xagmsg	32	1,440	544	Nadolny et al. 2023a
Taciba (SP)	-22.39000	-51.29000	annual crop	3	https://doi.org/10.15468/hfpvrg	28	1,260	165	Brown et al. 2023f
Londrina (PR)	-23.18527	-51.17833	annual crop	3	https://doi.org/10.15468/pucqj2	32	1,440	154	Brown et al. 2023j
Campo Mourão (PR)	-24.09000	-52.36000	annual crop	4	https://doi.org/10.15468/gxz6q2	16	720	0	Brown et al. 2023l
São Jerônimo da Serra (PR)	-23.730034	-50.731778	Organic annual crop	2	https://doi.org/10.15468/fxgq4p	16	720	240	Brown et al. 2023b
Jataizinho (PR)	-23.31472	-50.87028	Organic annual crop	2	https://doi.org/10.15468/dfdh2j	14	630	155	Brown et al. 2023a
Campo Mourão (PR)	-24.09000	-52.36000	annual crop	4	https://doi.org/10.15468/595jyb	16	720	0	Brown et al. 2023k
Londrina (PR)	-23.44333	-51.25805	annual crop / native vegetation	2	https://doi.org/10.15468/9ze9v2	16	720	180	Brown et al. 2023e
Sertanópolis (PR)	-23.16611	-51.16388	native vegetation	1	https://doi.org/10.15468/6r73ze	17	765	204	Brown et al. 2023g
Cornélio Procópio (PR)	-23.20000	-50.63000	Native vegetation / annual crop	2	https://doi.org/10.15468/qfzdte	13	540	144	Korasaki et al. 2023a
Londrina (PR)	-23.17166	-51.16583	annual crop	2	https://doi.org/10.15468/hcj7af	14	630	168	Brown et al. 2023c
Florínia (SP)	-22.88000	-50.73000	annual crop	2	https://doi.org/10.15468/6byztf	10	450	150	Brown et al. 2023d
Londrina (PR)	-23.20327	-51.17594	Organic crops	2	https://doi.org/10.15468/hya4u8	10	450	110	Brown et al. 2023h
Colombo (PR)	-25.31554	-49.15531	native vegetation / forestry plantation	7	https://doi.org/10.15468/w6v87y	8	360	40	Sautter et al. 2023
Londrina (PR)	-23.44277	-51.25222	native vegetation	1	https://doi.org/10.15468/43j5fq	7	315	77	Brown et al. 2023i
**23**				**122**		**1,855**	**83,085**	**25,921**	

**Table 3.** List of soils-related variables included in the "Extended measurement or fact" datasheets (results of soil chemical and physical analyses and measurements) for each of the 29 datasets available for download from GBIF (see links in Table 2).

**Variables**	**Unit**	**Description**
soil pH	-	soil potential hydrogen content
exchangeable aluminium	cmol_c_ dm^-3^	soil exchangeable aluminium content
potential acidity	cmol_c_ dm^-3^	soil potential acidity at pH 7
exchangeable potassium	cmol_c_ dm^-3^	soil exchangeable potassium content
exchangeable calcium	cmol_c_ dm^-3^	soil exchangeable calcium content
exchangeable magnesium	cmol_c_ dm^-3^	soil exchangeable magnesium content
available phosphorus	g kg^-1^	soil available phosphorus content
soil organic carbon	mg kg^-1^	soil organic carbon content
sum of bases	cmol_c_ dm^-3^	sum of calcium, magnesium and potassium contents
cation exchange capacity	cmol_c_ dm^-3^	soil cation exchange capacity at pH 7
base saturation	%	base saturation
soil organic nitrogen	g kg^-1^	soil organic nitrogen content
clay content	g kg^-1^	soil mineral particles content with size < 0.002 mm
silt content	g kg^-1^	soil mineral particles content ranging from 0.002-0.05 mm
sand content	g kg^-1^	soil mineral particles content ranging from 0.05-2.0 mm
bulk density	g cm^-3^	soil bulk density
moisture	%	gravimetric soil water content
