# Supplementary material for: Soil macrofauna communities in Brazilian land-use systems
Source: Biodivers Data J. 2024 Jan 15;12:e115000. doi: 10.3897/BDJ.12.e115000 (PMC10837794; doi:10.3897/BDJ.12.e115000)
Supplement: Supplementary material 1 — Table showing additional sites for which soil macrofauna data are available in Brazil [file bdj-12-e115000-s001.pdf]

No.	Counties (states)	Biome (IBGE, 2019)	Latitude	Longitude	Land use systems	No. sites, plots treatments	Records	Occurrence	Soils
1	Morretes (PR)	Atlantic Forest	-25.47922	-48.83323	native vegetation	1	36	1,620	504
2	Teresina (PI)	Cerrado	-5.038931	-42.80412	agroforestry	1	3	135	0
3	Ibaiti (PR)	Atlantic Forest	-23.7923042	-50.653895	pasture	1	9	405	144
4	Manaus<br>Teotônio (AM)	Amazonia	-3.107861111<br>-8.877638889	-59.91166667<br>-64.06227778	native vegetation	15	60	2,700	1,260
5	Anápolis (GO)<br>Campinas (SP)<br>Santo Antônio de Goiás (GO)<br>Londrina (PR)<br>Planaltina (PR)<br>Ponta Grossa (PR)<br>Sete Lagoas (MG)	Cerrado<br>Atlantic Forest<br>Cerrado<br>Atlantic Forest<br>Cerrado<br>Atlantic Forest<br>Cerrado	-16.32<br>-22.6<br>-16.46666<br>-23.195833<br>-15.45<br>-25.16<br>-19.45	-48.94<br>-46.91<br>-49.28333<br>-51.18<br>-47.61<br>-50.08<br>-44.17	annual crops	57	228	10,260	0
6	Londrina (PR)	Atlantic Forest	-23.331285	-51.198512	annual crops	5	36	1,620	0
7	Rio Claro (SP)	Cerrado	-22.50897	-47.613	perennial crops, native forest, pasture	3	18	810	126
8	Rio Verde (GO)	Cerrado	-17.51748	-51.39366	native vegetation, annual crops	7	62	2,790	0
9	São Jerônimo da Serra (PR)<br>Alvorada do Sul (PR)<br>Janiópolis (PR)	Atlantic Forest	-23.721424<br>-22.821491<br>-24.1411	-50.758206<br>-51.253196<br>-52.778851	perennial crops	18	135	6,075	1,890
10	Brasília (DF)	Cerrado	-15.952666	-47.876666	native vegetation	10	40	1,800	200
11	Manaus (AM)	Amazonia	-2.5166666	-60.016666	agrosilviculture, pasture, native forest	4	43	1,935	0
12	Santa Helena de Goiás (GO)	Cerrado	-17.71666	-50.58333	annual crops, native vegetation	5	17	765	0
13	Xanxerê (SC)<br>Chapecó (SC)	Atlantic Forest	-26.3565<br>-27.1935	-52.481944<br>-52.65933	annual crops, native vegetation	10	60	2,700	780
14	Redenção (CE)	Caatinga	-4.248055	-38.75277	annual crops, native vegetation, agroforestry, pasture	6	60	2,700	440

15	Bituruna (PR)	Atlantic Forest	-26.1578	-51.5548	annual crops, native vegetation	9	60	2,700	0
16	Iranduba (AM)<br>Belterra (PA)<br>Porto Velho (RO)	Amazonia	-3.24640945<br>-2.78480898<br>-8.87661786	-60.22552<br>-54.99786<br>-64.06662	annual crops, native vegetation, pasture	18	90	4,050	810
17	Quitandinha (PR)	Atlantic Forest	-25.890771	-49.455284	annual crops, native vegetation, horticulture	4	36	1,620	540
18	Ponta Grossa (PR)	Atlantic Forest	-25.12497	-50.05125	annual crops, agrosilvopastoral, agropastoral, native vegetation, pasture, forestry plantation	7	105	4,725	1,575
19	Quixeramobim (CE)	Caatinga	-5.12002	-39.17591	native vegetation	2	30	1,350	30
20	Guarapuava (PR)	Atlantic Forest	-25.56126	-51.48983	native vegetation, annual crops	6	18	810	198
21	Campos Novos (SC)<br>Vacaria (RS)<br>Zortéa (SC)	Atlantic Forest	-27.49302<br>-28.59276<br>-27.43772	-51.44327<br>-51.03484<br>-51.45405	annual crops, native vegetation	12	108	4,860	0
22	Chapecó (SC)	Atlantic Forest	-27.22037	-52.82716	annual crops, native vegetation	4	32	1,440	480
23	Bocaina de Minas (MG)	Atlantic Forest	-22.19817	-44.60176	agroforestry, orchard, pasture, native vegetation	12	72	3,240	1,008
24	São Sebastião do Paraíso (MG)	Cerrado	-20.92048	-46.98456	perennial crop	2	10	450	80
25	Bananal (SP)<br>Itaberá (SP)<br>Itapeva (SP)<br>Barra do Chapéu (SP)<br>Iporanga (SP)	Atlantic Forest	-22.80722<br>-23.83916<br>-24.071944<br>-23.84083<br>-24.47777	-44.36638<br>-49.14444<br>-49.01861<br>-49.14444<br>-49.60388	native vegetation, forestry plantation	12	180	8,100	4,320
26	Capão do Leão (RS)	Pampa	-31.80152	-52.41538	fallow, annual crops	5	40	1,800	280
27	Dois Vizinhos (PR)	Atlantic Forest	-25.69397	-53.1032	native vegetation, regeneration	32	32	1,440	216
28	Santa Maria (RS)	Pampa	-29.69305	-53.79204	forestry plantation, fallow, native grass pasture	5	25	1,125	200
29	Santa Maria de Jetibá (ES)<br>Domingos Martins (ES)<br>Marechal Floriano (ES)	Atlantic Forest	-20.07416<br>-20.50888<br>-20.64472	-40.78611<br>-41.06750<br>-44.04361	perennial crop	12	108	4,860	1,512

30	Nova Ipixuna (PA)	Amazonia	-4.80596	-49.36581	native forest, pasture	18	90	4,050	1,800
31	Sebastião Leal (PI)<br>Uruçuí (PI)<br>Bom Jesus (PI)<br>Baixa Grande do Ribeira (PI)	Cerrado	-7.65388<br>-7.23527<br>-9.17638<br>-7.80277	-44.04361<br>-44.63583<br>-44.84333<br>-45.01666	annual crops, native vegetation	12	60	2,700	180
32	Campo Belo do Sul (SC)<br>Santa Teresinha do Salto (SC)<br>Otacílio Costa (SC)<br>Xanxerê (SC)<br>São Miguel do Oeste (SC)<br>Chapecó (SC)<br>Joinville (SC)<br>Blumenau (SC)<br>Timbó (SC)<br>Orleans (SC)<br>Siderópolis(SC)<br>Lauro Muller (SC)	Atlantic Forest	-27.88203<br>-27.79965<br>-27.59435<br>-26.8337<br>-26.73815<br>-27.0343<br>-26.21996<br>-26.77835<br>-26.79738<br>-28.37729<br>-28.58239<br>-28.35479	-50.65555<br>-50.59563<br>-49.84868<br>-52.453<br>-53.53495<br>-52.6965<br>-48.85539<br>-49.09824<br>-49.26532<br>-49.26040<br>-49.40531<br>-49.43559	native vegetation, forestry plantation, pasture, agropastoral, annual crops	120	1080	48,600	20,520
33	Melgaço (PA)	Amazonia	-1.73762	-51.44566	native vegetation	40	240	10,800	2,640
34	Nova Brasilândia d'Oeste (RO)	Amazonia	-11.72568	-61.77807	orchard	5	10	450	110
35	Itinga (MA)<br>Alcântara (MA)<br>São Luís (MA)<br>Tomé-Açu (PA)<br>São José de Ribamar (MA)<br>Rosário (MA)<br>Centro Novo (MA)	Amazonia	-3.91765<br>-2.41761<br>-2.58750<br>-2.64387<br>-2.86122<br>-3.68787	-46.76388<br>-44.42221<br>-44.21194<br>-44.13823<br>-44.13344<br>-46.75965	agroforestry, native vegetation	85	439	19,755	0
36	Macapá (AP)	Amazonia	0.38	-51.03	annual crops	1	16	720	224
37	Macapá (AP)	Amazonia	0.96	-50.96	annual crops	6	18	810	180
38	Macapá (AP)	Amazonia	-0.051	-51.12	native vegetation	22	22	990	286
39	Laranjal do Jari (AP)	Amazonia	-0.5641	-52.3065	native vegetation	22	22	990	198

40	Tomé Açu (PA)	Amazonia	-2.40379	-48.24761	orchard, native vegetation, pasture	3	27	1,215	0
41	Jaboti (PR)	Atlantic Forest	-23.70289	-50.12447	perennial crops	4	40	1,800	320
42	Jardim Olinda (PR)	Atlantic Forest	-22.57002	-52.05663	agrosilvopastoral	8	40	1,800	520
43	São Gabriel (RS)	Pampa	-30.43048	-54.36829	native vegetation, forestry plantation	3	21	945	294
44	Correntina (BA)	Cerrado	-13.78361	-46.00361	anual crops, pasture, native vegetation	6	30	1,350	360
45	Planaltina (DF)	Cerrado	-15.58333	-47.86666	annual crops, pasture, native vegetation	13	36	1,620	252
46	Pentecoste (CE)	Caatinga	-3.81880	-39.34003	native vegetation	2	56	2,520	672
47	Manduri (SP)<br>Brotas (SP)<br>Caiuá (SP)<br>Rancharia (SP)<br>Naviraí (MS)<br>Ipameri (GO)	Cerrado	-22.28333<br>-23.00000<br>-23.60730<br>-22.14872<br>-22.99402<br>-17.65750	-48.11666<br>-49.31666<br>-49.86608<br>-49.81425<br>-54.38022<br>-48.20611	native vegetation, pasture, perennial crops, agropastoral, agrosilvopastoral	25	180	8,100	1,800
48	Brasília (DF)	Cerrado	-15.61222	-47.75525	pasture	12	36	1,620	144
49	Nova Friburgo (RJ)	Atlantic Forest	-22.28750	-42.66016	annual crops, fallow, horticulture	5	25	1,125	250
50	Humaitá (AM)	Amazonia	-7.57901	-63.14831	native vegetation	3	26	1,170	0
51	São Gabriel da Cachoeira (AM)	Amazonia	0.21166	-66.76333	native vegetation	5	25	1,125	350
52	Chapecó (SC)<br>Pinhalzinho (SC)<br>São Miguel do Oeste (SC)	Atlantic Forest	-27.07536<br>-26.87325<br>-26.73905	-52.68363<br>-52.95758<br>-53.54225	native vegetation, annual crops, pasture, fallow, forestry plantation	27	174	7,830	1,740
53	Maracajú (MS)	Cerrado	-21.61444	-55.16833	agropastoral, native vegetation	7	48	2,160	720
54	Esperantina (PI)	Cerrado	-3.90194	-42.23388	agroforestry, native vegetation, annual crops	10	50	2,250	0
55	Manaus (AM)	Amazonia	-2.80305	-60.15555	native vegetation	10	50	2,250	630
56	Pirapemas (MA)	Cerrado	-3.73083	-45.39861	native vegetation	12	192	8,640	1,536
57	Lauro Müller (SC)	Atlantic Forest	-28.36616	-49.45860	forestry plantation, pasture, native vegetation, annual crop	18	78	3,510	702
58	Campinas (SP)	Atlantic Forest	-22.90555	-47.09277	annual crops, fallow	8	64	2,880	640

59	Seropédica (RJ)	Atlantic Forest	-22.75833	-43.68611	orchard	9	9	405	45
60	Belém (PA)	Amazonia	-4.25	-8.75	native vegetation	4	40	1,800	1,120
61	São Paulo (SP)	Atlantic Forest	-23.65090	-46.62252	native vegetation	6	54	2,430	756
62	Manaus (AM)	Amazonia	-2.98403	-60.20130	orchard, native vegetation	10	30	1,350	300
63	Dourados (MS)	Atlantic Forest	-22.27753	-54.81290	annual crops, agropastoral, pasture, native vegetation	8	40	1,800	0
64	Bom Jardim (RJ)	Atlantic Forest	-22.16722	-42.28722	annual crops, fallow, native forest	30	186	8,375	2,232
65	Valença (RJ)	Atlantic Forest	-22.37277	-43.78972	pasture, native forest	6	79	3,555	474
66	Paraty (RJ)	Atlantic Forest	-23.16722	-44.73694	perennial crops, pasture, annual crops, native vegetation	12	111	4,995	0
67	Itaboraí (RJ)	Atlantic Forest	-22.75544	-42.85694	native vegetation, pasture	8	120	5,400	1,560
Total	**55**					**834**	**1,762**	**230,445**	**55,882**
